# Investigating the effects of a novel gamified cognitive training on adolescent mental health

**DOI:** 10.1186/s13034-025-00917-1

**Published:** 2025-07-03

**Authors:** Karina Grunewald, Savannah Minihan, Jack L. Andrews, Annabel Songco, Sarah-Jayne Blakemore, Anson Kai Chun Chau, Jaimee Fischer, Elaine Fox, Alba Bruggeman Nelissen, William Raffe, Matthew Richards, Aliza Werner-Seidler, Susanne Schweizer

**Affiliations:** 1 School of Psychology, University of New South Wales, Sydney, Australia; 2 Department of Experimental Psychology, University of Oxford, Oxford, UK; 3 Black Dog Institute, University of New South Wales, Sydney, Australia; 4 Department of Psychology, University of Cambridge, Cambridge, UK; 5 School of Psychology, University of Adelaide, Adelaide, Australia; 6 Faculty of Psychology and Educational Sciences, Catholic University of Leuven, Leuven, Belgium; 7 Center for Human Centred Technology Design, University of Technology Sydney, Sydney, Australia

**Keywords:** Mental health, Adolescent, Depression, Emotion regulation, Cognitive training, Gamification

## Abstract

**Background:**

Adolescence is a time of increased emotional volatility, with emotion regulation still developing. Training the cognitive substrate of successful emotion regulation has been shown to benefit adolescents’ mental health. However, cognitive training interventions often have low adherence rates in this age group. The current study therefore trialled a novel gamified cognitive training program in adolescents.

**Methods:**

A longitudinal study was conducted throughout 2023 where 144 culturally diverse adolescents (13–16 years, 48% female) completed 12 days of either a novel gamified affective control training program, the Social Brain Train (SBT), or a standard non-gamified affective control training program (AffeCT). Participants also completed mental health and mechanisms of change questionnaires at baseline, post-training, and 1-month follow-up, as well as behavioural affective control and interpretation bias measures at baseline and post-training.

**Results:**

The total minutes spent training did not differ significantly across the two training groups. Participants assigned to SBT training, however, did engage in more training sessions than participants assigned to AffeCT training. Additionally, all participants showed improvements in affective control performance and a reduction in interpretation bias and rumination from baseline to post-training. The observed reduction in rumination persisted at 1-month follow-up.

**Conclusions:**

As engagement is often the most difficult thing to achieve in cognitive training with adolescents, observing greater repeated engagement with the gamified cognitive training is promising, given training on these apps is entirely self-motivated. Observing benefits to affective and cognitive control performance as well as reduced interpretation bias and rumination tendencies after very limited training is promising, as these factors have all been previously linked to improved mental health symptoms among adolescents. The present findings therefore suggest there may be merit in using gamification techniques to improve the design of future training programs, and employing these to improve affective, cognitive, and emotion regulation abilities in adolescents.

**Supplementary Information:**

The online version contains supplementary material available at 10.1186/s13034-025-00917-1.

## Introduction

 Adolescence (10–24 years; [1, 2]), is a sensitive period for the development of mental health disorders. Indeed, most mental health disorders first onset by 24 years, and often persist across the lifespan [3]. Of these, emotional disorders, including depression and anxiety, are the most common [4]. Yet, despite these disorders being the leading burden of disease in adolescence [5, 6], current psychological and pharmacological treatments often show limited efficacy [7, 8]. Meta-analytic evidence suggests that less than 40% of adolescents respond to psychological treatments for depression [9], and only 36% of adolescents receiving treatment for anxiety disorders were in remission post treatment [10]. Adolescent responses to pharmacological treatments of depression have been similarly low [11]. Identifying novel avenues for intervention and prevention of adolescent anxiety and depression that are deliverable at scale is therefore urgent.

Researchers have proposed that current interventions may be unsuccessful in treating emotional disorders, as they fail to target key risk factors underlying vulnerability to these disorders [12]. One such risk factor is emotion regulation [13], individuals’ ability to influence their emotions, where emotions refer to temporally limited, positively or negatively valenced and situationally-bound experiences [14]. Difficulties in emotion regulation during adolescence have been robustly linked to the onset and maintenance of adolescent mental health problems [15], as well as treatment responsiveness [16]. Developmental research shows that emotion regulation improves throughout adolescence [17], in tandem with its underlying cognitive [18] and neural [19] substrates. Successful emotion regulation has been proposed to rely on cognitive control [20], the ability to attend to and respond to goal-relevant stimuli while inhibiting attention and responses to goal-irrelevant stimuli [21]. Especially, improvements in the application of cognitive control in affective contexts, *affective control*, may be central to the development of successful emotion regulation [22]. Targeting these cognitive substrates has therefore been proposed as an effective means to intervene in the development and maintenance of emotional disorders [23].

Encouragingly, researchers have found that affective control is amenable to cognitive training, and training effects have been associated with improvements in emotion regulation and a range of clinical outcomes including depression and anxiety in adolescence [23]. However, studies have found that cognitive training interventions have low engagement and adherence rates among adolescents [24–26], as adolescents often report finding low motivation for exerting effort to complete cognitive tasks [27]. As a result, while improving affective control through cognitive training constitutes a promising avenue for intervention, low engagement limits its viability as an intervention or prevention.

One way to effectively augment adolescents’ participation in cognitive training is *gamification*. Gamification, the use of game-like features such as narrative storylines and points systems, has been reliably shown to improve both attentional engagement with training and motivation to increase training time [28]. It may be especially useful for interventions aimed at adolescents, as 90% of Australian adolescents report playing digital games regularly, averaging approximately 98 min of gameplay each day [29]. Gamification also allows the creation of ecologically-valid content that is reflective of users’ real-world environments and experiences. As motivation and attentional capture are crucial for cognitive training success [30], and ecological validity improves the transferability of training gains to real-world problems [31], gamification appears to offer an existing opportunity to boost the uptake of cognitive training in adolescents.

The current study aimed to explore the effectiveness of a novel gamified app-based affective control training program, the Social Brain Train (SBT), and to assess whether gamification would lead to improved training uptake and outcomes. The SBT comprised three components: a standard affective control training component (affective dual *n*-back task), a cognitive interpretation bias modification (CBM-I) component and a psychoeducation component. The affective dual *n*-back task required flexible engagement (remembering emotional words) and disengagement (inhibiting the processing of the task-irrelevant emotional expressions) with affective information. This specific task has previously been shown to lead to improvements in affective control, emotion regulation, and mental health in adolescents and young adults [26, 32, 33]. In the SBT, the affective *n*-back task was gamified, with points awarded for performance.

The CBM-I component required participants to solve ambiguous social “puzzles” in a positive way. CBM-I training has previously been shown to successfully reduce interpretation bias in adolescents [34], which is associated with adolescent depression and anxiety [4, 35]. The CBM-I also introduced a new “game” element to the training and allowed participants to directly train the application of affective control, as adolescents had to override any prepotent negative response tendencies and regulate any affective responses that the scenarios may elicit to resolve the puzzles positively.

Lastly, the SBT further promoted gamification through the inclusion of badges and fun facts about mental health and the human brain (i.e., psychoeducation component) that could be unlocked throughout training completion. Psychoeducation in itself has small but positive effects on emotional disorders [36, 37]. Combining affective control training with CBM-I then increases opportunities to practice the application of affective control in real-world analogue scenarios. This intervention’s optimisation was further enhanced by psychoeducation contents that provide a rationale to motivate participation in the training.

The SBT’s effectiveness was compared with a non-gamified affective control training (AffeCT) to investigate the impacts of gamification on training. Participants were 13–16 years of age, as this age range precedes and includes the ages – 16–17 years [38] – at which a sudden rise in symptoms of emotional disorders is observed in epidemiological studies. The present study allowed us to investigate the following pre-registered hypotheses:

1. The SBT group would spend more time training (in minutes) than the AffeCT group.
2. Time spent training across groups would be associated with improved performance on a non-trained affective control task (H2a) and social interpretation bias (H2b); and the effect of training time on interpretation bias would be greater in the SBT than the AffeCT group (H2c).
3. Improvements in affective control would be associated with baseline to post-training changes in emotion regulation (H3a) and improvements in affective control and interpretation bias would be associated with lower depressive and anxiety symptoms (H3b).

## Materials and methods

This study was approved by the University of New South Wales Human Research Executive Committee (HC230164) and a protocol was pre-registered prior to participant recruitment (https://osf.io/preprints/psyarxiv/rpvh9). 

### Sample size calculation

Assuming a moderate effect of training on mental health (Cohen’s *f* = 0.20) required t a sample of *N* = 102 to achieve a power of 90% with significance thresholded at α = 0.008 to correct for 6 outcomes of interest. While a review of paediatric cognitive control training showed moderate to large effects of training on various clinical outcomes [23], we used the small to moderate effect size listed above to be conservative as some of our outcomes of interest had not previously been tested using affective control training. Assuming an attrition/non-compliance rate of 40% [24], we aimed to recruit *N* = 144 participants who completed the study faithfully (failed no more than 2 out of 5 attention check items at baseline).

### Eligibility criteria

To sign up for the study, participants had to: be aged 13–16 years; have daily access to a device with internet connectivity (e.g., smartphone or tablet), as the experiment was conducted completely online; read English with native fluency, as all assessments and training relied on adequate reading ability; and have no history of traumatic brain injury (TBI), as the training placed significant demands on executive functioning, which is often impaired in those with TBI [39].

### Participant exclusions

In total, 253 participants were recruited and completed the baseline questionnaires (Figure S1). Of these, 1 participant withdrew from the study and 12 failed more than 2 attention checks and were excluded from the study resulting in a total of 240 participants recruited and completing baseline questionnaires. One additional participant failed more than 2 attention checks at baseline, but due to experimenter error was still assigned to a training app and completed training, so their baseline data was excluded but their training, post-training and follow-up assessment data was retained.

96 participants were excluded from data analyses as they were flagged as likely fraudulent based on the following set of criteria (Figure S1): Qualtrics, the platform, which was used for study sign-ups has measures to indicate whether individuals completing the questionnaire are likely to be fraudulent or duplicates of previous participants; participants with duplicate scores higher than 75 or fraud scores higher than 30 on Qualtrics were excluded, as per the platform’s guidelines [40]. Additionally, after the experiment’s advertisement had been online for over two weeks and signups had dwindled to only a few each day, experimenters also began to observe “bulk” signups, where multiple participants (10+) signed up within a few minutes of each other, most with high duplicate/fraud scores. However, these “fraudulent” participants were still able to complete the study and received remuneration for their participation, as we were ethically bound to provide this once they were included in the study. The final sample used for data analyses from those recruited was therefore *N* = 144 (Table 1). While here we report the analyses with only those participants who appeared legitimate, we additionally repeated all data analyses with “fraudulent” participants included (see Tables S12-15 for output of the pre-registered analyses with the full sample included). The pattern of results was similar across both samples.

### Participants

As mentioned above, the final sample size used for data analysis comprised 144 participants recruited in 2023 through schools, social media advertisement, the MQ participate and Children Helping Science websites, and advertisements on the research lab website and the community (Table 1). Where possible, advertisements and invitations were targeted at individuals residing in Australia and the United Kingdom (UK), where the lead researchers were based. However, some participants from the USA and India signed up for the study through online recruitment platforms. If these participants obtained parental consent and met all other eligibility requirements, they were included in the study.

**Table 1:** Sample characteristics (*N* = 144), means and standard deviations of variables

	Mean (SD)/*N* (%)
Age (years)	14.77 (1.12)
SES	2.81 (0.31)
Gender	
Female	69 (47.92%)
Male	73 (50.69%)
Non-binary	2 (1.39%)
Country	
Australia	77 (53.47%)
United Kingdom	48 (33.33%)
United States of America	17 (11.81%)
India	2 (1.39%)
Ethnicity	
Aboriginal or Torres Strait Islander	2 (1.39%)
Asian	22 (15.28%)
Black	7 (4.86%)
White	98 (68.06%)
Mixed	10 (6.94%)
Other	4 (2.78%)
Prefer not to say	1 (0.69%)
Education	
Current student	142 (%)
Prefer not to say	2 (%)

*SES* = socio-economic status, derived from the average level of parental education for up to 2 parents (1 = primary school; 2 = high school, professional/vocational training; 3 = university)

### Consent and assent

Before completing the study, prospective participants provided informed consent from their parent or guardian. Parents/guardians could follow a URL to access study information and the consent procedure on Qualtrics (https://www.qualtrics.com). Eligible participants with parental/guardian consent were invited to complete an informed assent procedure on Gorilla Experiment Builder (https://gorilla.sc), after which they were given access to the baseline assessment. During baseline and post-training assessments, participants were reminded that they could withdraw consent at any time before, during, or after the study with no consequences, and that they would receive up to AUD60 (GBP30) reimbursement for all parts of the study completed up to withdrawal.

### Allocation procedure

Eligible participants who completed the baseline questionnaires were randomised to either the SBT (*N* = 77) or the AffeCT (*N* = 67) group. Randomization, performed through computer-generated group assignment [41], was stratified by age and based on block randomization sequence with randomly mixed block sizes [2–6]. JF and ABN were assigned to conduct group allocation and baseline assessments and to answer any participant queries, concerns or issues throughout study completion, and were not involved in data analysis. The remaining experimental staff were blinded to group allocation and helped conduct the remaining participant testing.

### Measures

#### Participant characteristics

A brief questionnaire was used to measure demographic characteristics (e.g., age, gender, ethnicity), history of mental health and learning and neurodevelopmental disorders. To explore whether training increased overall screentime use, participants were also asked for their average daily screentime usage (indicated within their device settings). These measures were all assessed at baseline, with screentime assessed again at post-training.

#### Mental health and functioning

**Anxiety symptoms.** Symptoms of anxiety were measured at baseline, post-training, and follow-up with the Generalized Anxiety Disorder 7 Scale (GAD-7; 42). The scale was made up of 7 items assessing how often anxiety symptoms were experienced over the last two weeks on a 4-point Likert scale ranging from 0 (*not at all*) to 3 (*nearly every day*). The scale demonstrated good internal consistency in the current sample at all time points (baseline: ω_T_ = 0.94; post-training: ω_T_ = 0.94; follow-up: ω_T_ = 0.96). Item scores were summed, with higher scores indicating greater severity of anxiety symptoms.

**Depression symptoms.** Symptoms of depression were measured at baseline, post-training, and follow-up with the Patient Health Questionnaire– Adolescent (PHQ-A; 43), a scale made up of 8 items assessing severity of depressive symptoms over the last two weeks. The 9th item, measuring suicidality, was omitted from the present study, as risk could not be managed in the context of an online study. Items were rated from 0 (*not at all*) to 3 (*nearly every day*) and were then summed, with higher scores indicating greater severity of depression symptoms. Internal consistency for the PHQ-A was excellent at each timepoint for the current sample (baseline: ω_T_ = 0.94; post-training: ω_T_ = 0.95; follow-up: ω_T_ = 0.96).

**Functional impairment.** The functional impact of negative feelings on individuals’ functioning was assessed at baseline, post-training, and follow-up with a modified version of the Child Anxiety Life Interference Scale (CALIS; 44). The scale comprised 9 items measuring to what extent negative feelings upset participants or prevented them from doing a range of activities (e.g., getting on with parents, completing schoolwork, playing sport). Items were rated on a 5-point Likert scale ranging from *not at all* to *a great deal* and were then summed such that higher scores indicated greater functional impact of negative feelings. The CALIS showed good internal consistency in the current sample at all time points (baseline: ω_T_ = 0.90; post-training: ω_T_ = 0.90; follow-up: ω_T_ = 0.93).

#### Mechanisms of change

**Emotion regulation.** Emotion regulation was measured at baseline, post-training, and follow-up with the reappraisal subscale of the Emotion Regulation Questionnaire– child and adolescent version (ERQ-CA; 45), a 10-item scale measuring tendency to regulate emotions through cognitive reappraisal (e.g., “I control my feelings about things by changing the way I think about them”) and expressive suppression (e.g., “I control my feelings by not showing them”). Items were rated on a 7-point Likert Scale ranging from 1 (*strongly disagree*) to 7 (*strongly agree*). The reappraisal subscale was made up of 6 items, summed such that higher scores indicated greater emotion regulation, and showed good internal consistency at all time points (baseline: ω_T_ = 0.90; post-training: ω_T_ = 0.93; follow-up: ω_T_ = 0.91).

**Rumination.** Rumination was assessed at baseline, post-training, and follow-up with the Repetitive Thinking Questionnaire (RTQ-10; 46). The scale was made up of 10 items rated on a 5-point Likert scale ranging from 1 (*not true at all*) to 5 (*very true*). Items measured how participants reacted in distressing situations (e.g., “I think about the situation all the time”), and scores were summed such that higher scores indicated greater rumination. The scale showed good internal consistency at all time points (baseline: ω_T_ = 0.92; post-training: ω_T_ = 0.93; follow-up: ω_T_ = 0.93).

**Social sensitivity.** Social sensitivity was assessed at baseline, post-training, and follow-up with the Online and Offline Social Sensitivity Scale (O2S3; 47). The O^2^S^3^ assessed social sensitivity in both offline and online contexts through 18 items rated on a 4-point Likert scale ranging from 0 (*strongly disagree*) to 3 (*strongly agree*). Scores were summed such that higher scores indicated greater social sensitivity. The scale demonstrated acceptable internal consistency at all time points (baseline: ω_T_ = 0.90; post-training: ω_T_ = 0.92; follow-up: ω_T_ = 0.87).

**Social risk concern.** Concern for social risk was measured at baseline, post-training, and follow-up with the Health and Social Risk Questionnaire’s (HSRQ; 48) social subscale. This subscale was made up of 6 items assessing how worried participants would feel performing different actions relating to social activities (e.g., “defend an unpopular opinion that you believe in”). The items were rated on a rating scale ranging from 0 (*not worried at all*) to 100 (*very worried*). Social risk concern was indexed as the average of these items, with higher scores indicating greater concern. The scale demonstrated acceptable internal consistency at all time points (baseline: ω_T_ = 0.78; post-training: ω_T_ = 0.84; follow-up: ω_T_ = 0.80).

**Interpretation bias.** Interpretation bias was measured at baseline and post-training through a scrambled sentences task (SST) developed for adolescents [49]. The task comprises 40 trials reflecting general and social anxiety-related concerns. Twenty trials were completed at baseline, and 20 at post-training (counterbalanced across participants). Each trial required participants to unscramble a scrambled statement. Statements could be unscrambled to hold a positive or negative meaning. Each trial required participants to include five of six available words in a statement within 30 s. For example, “people, dislike, new, enjoy, meeting, I” could be unscrambled to “I enjoy/dislike meeting new people”, with the selection of enjoy or dislike rendering the statement positive or negative, respectively. In the present study, the statement “relaxed with tense I’m *children older*” was modified to “relaxed with tense I’m *people other*”, to ensure all sentences were age appropriate for our sample. Additionally, each administration of the task contained one neutral sentence (e.g., “I read like books to magazines”) as a baseline response for the task. That is, the current version included twenty-one trials at baseline and post-intervention.

The task was performed under a cognitive load, with participants required to hold a 4-digit number in mind throughout the task. The cognitive load was introduced to the task to disrupt volitional efforts to supress, modify or edit thoughts [49]. Interpretation bias was operationalised as the proportion of sentences completed correctly with a negative valence, such that higher scores indicated greater negative interpretation bias. The inclusion of the neutral sentences as a covariate in the analyses did not change the pattern of results and is therefore not reported in the current manuscript.

**Affective control.** Affective control was assessed at baseline and post-training with a 2-back task [50]. Participants indicated via button press whether the word they were currently seeing was the same as the word presented two-words-back. The task was completed twice, once with 22 neutral words (e.g., stair) and once with 22 affective words (e.g., rude) from the English lemmas database [51]. Trials (i.e., words) progressed if no response was given after 2,500 ms. Affective control was operationalised as average reaction time (RT) on correct trials of the affective minus neutral 2-back conditions, with more negative scores indicating greater affective control.

#### Acceptability

Intervention acceptability measures were adapted from an existing smartphone acceptability measure [52] and assessed at post-training. Participants were first asked how much of the training they had completed on their assigned app: none of it, part of it, most of it, or almost all of it. Those who selected an answer indicating at least partial training completion were then asked to answer whether they believed their assigned app was helpful, easy to use, and whether they liked using it. These three additional questions were rated on 5-point Likert Scales ranging from 0 (*not at all*) to 4 (*very*).

#### Training programs

Participants completed 12 training sessions of either SBT or AffeCT over a 15-day period. To promote training completion, participants received training reminders at 8am and 5pm each day. Training was self-paced, as total time training was one of the outcomes of interest. Once all 12 sessions were completed, participants could opt to repeat any previously completed session.

#### Social brain train

**Fig. 1:**
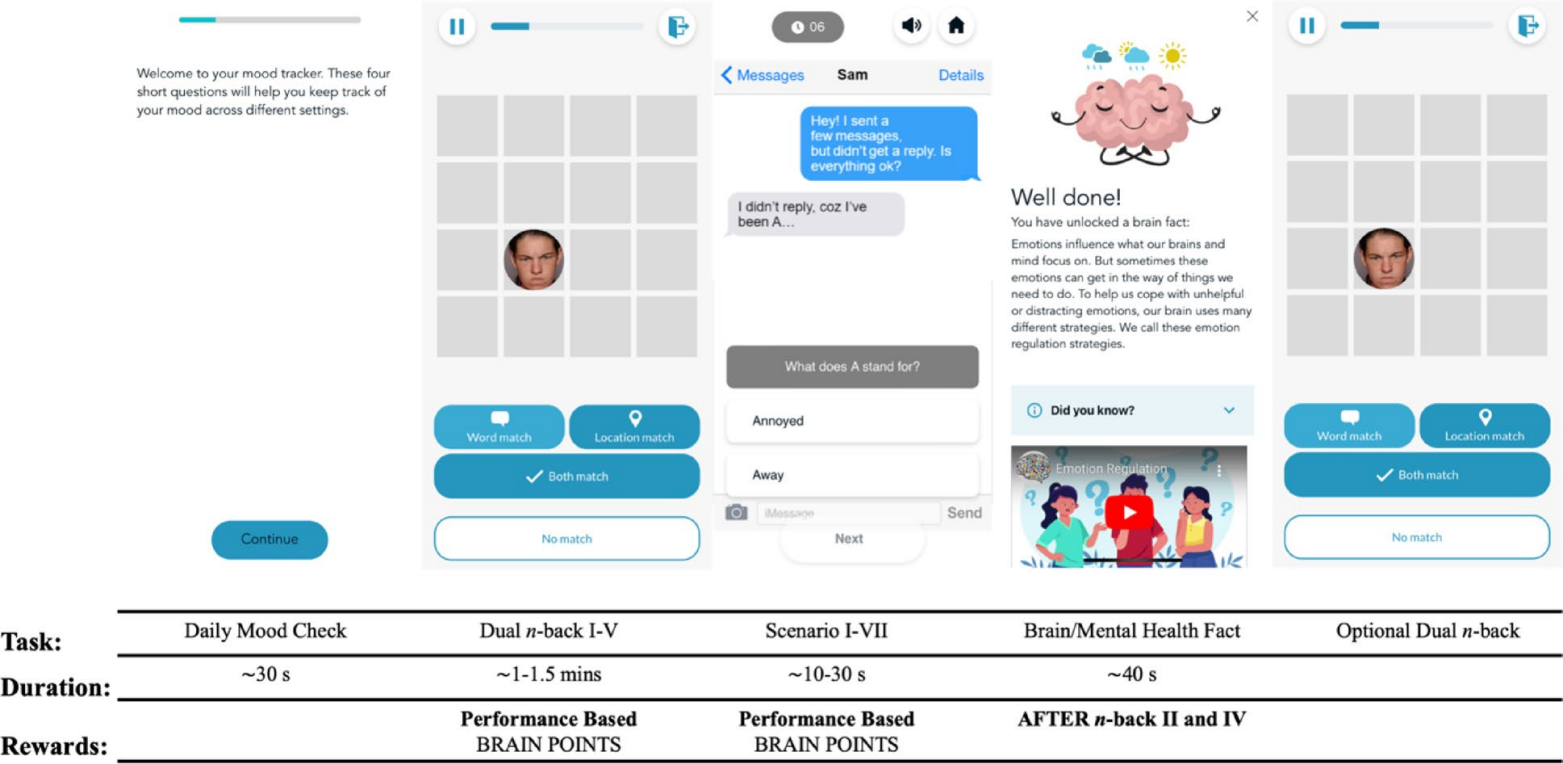
Depiction of one training session of the SBT *Note* Figure 1 shows the structure of one training session of the SBT. Participants first completed four brief questions (~ 30 s) about their mood, affect regulatory intentions, social context, and current activity. Participants then alternated between completing five blocks of affective control training (~ 1–1.5 min per block) and seven ambiguous social interaction scenarios (~ 10–20 s per scenario). Within each training session, participants unlocked facts about mental health and the social and emotional brain (~ 40 s per fact) after blocks two and four of affective control training. Participants also received points after completing each block of affective control training (1 point: ≤ 50% correct; 10 points: 51–80% correct; 30 points: ≥ 81% correct), and 2 additional points for each social interaction scenario completed correctly (i.e., scenarios where the positive resolution was selected). Upon session completion, the app flow locked and participants were unable to access the next training session until the following day, but could still complete as many extra blocks of affective control training as they wished.

In the SBT each training session began with four brief questions about participants’ mood (ranging from *very unhappy* to *very happy*), affect regulatory intentions (e.g., avoidance, acceptance), social context (e.g., alone, with friends/family), and current activity. Participants then alternated between the affective control (5 blocks), CBM-I (7 scenarios) and psychoeducation (1 brain and 1 mental health fact) components (Fig. 1). At the end of each training session, participants had the option to continue training on the affective control component, however the next training session could not be accessed until the following day.

**Fig. 2:**
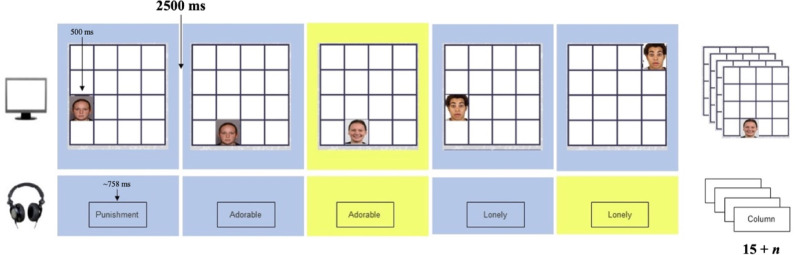
Depiction of the affective control training components *Note* Figure 2 depicts a block of 15 + n trials of the affective control training component of the SBT where *n* = 1. Participants were therefore asked to indicate whether the location of the face presented in the current trial matched the location of the face presented in the previous trial, and whether the word presented in the current trial was also presented in the previous trial. Target trials (i.e., trials with a location and/or a word match) are depicted with a yellow background. Participants indicated whether either or both stimuli matched the stimuli presented *n*-trials back within 2500 ms from the onset of the stimuli. After a response was made or the time expired, the next word-image pairing then appeared.

**Affective control training.** The affective dual *n*-back task comprised 15 + *n* trials where the image of a face (appearing on a 4 × 4 grid for 500 ms) and a spoken word were presented simultaneously (Fig. 2). Participants were asked to indicate whether the stimuli were the same (words) or appeared in the same location (faces) as the stimuli presented *n*-trials back (for details on stimuli, please see 53). Responses were indicated via button press (*no match*, *location match*, *word match*, or *both match*), with feedback provided after each trial. Participants had 2500 ms to respond before the next word/face pair appeared, with responses marked as incorrect if no selection was made within this time.

At the start of training (i.e., the first training session), *n* was set to one. For each subsequent training session, the starting level of *n* was the final *n* achieved the previous session minus two. The level of *n* within a training session was titrated to performance. When performance reached ≥ 70% accuracy, *n* increased by one, while *n* decreased by one when accuracy was ≤ 30%.

**Fig. 3:**
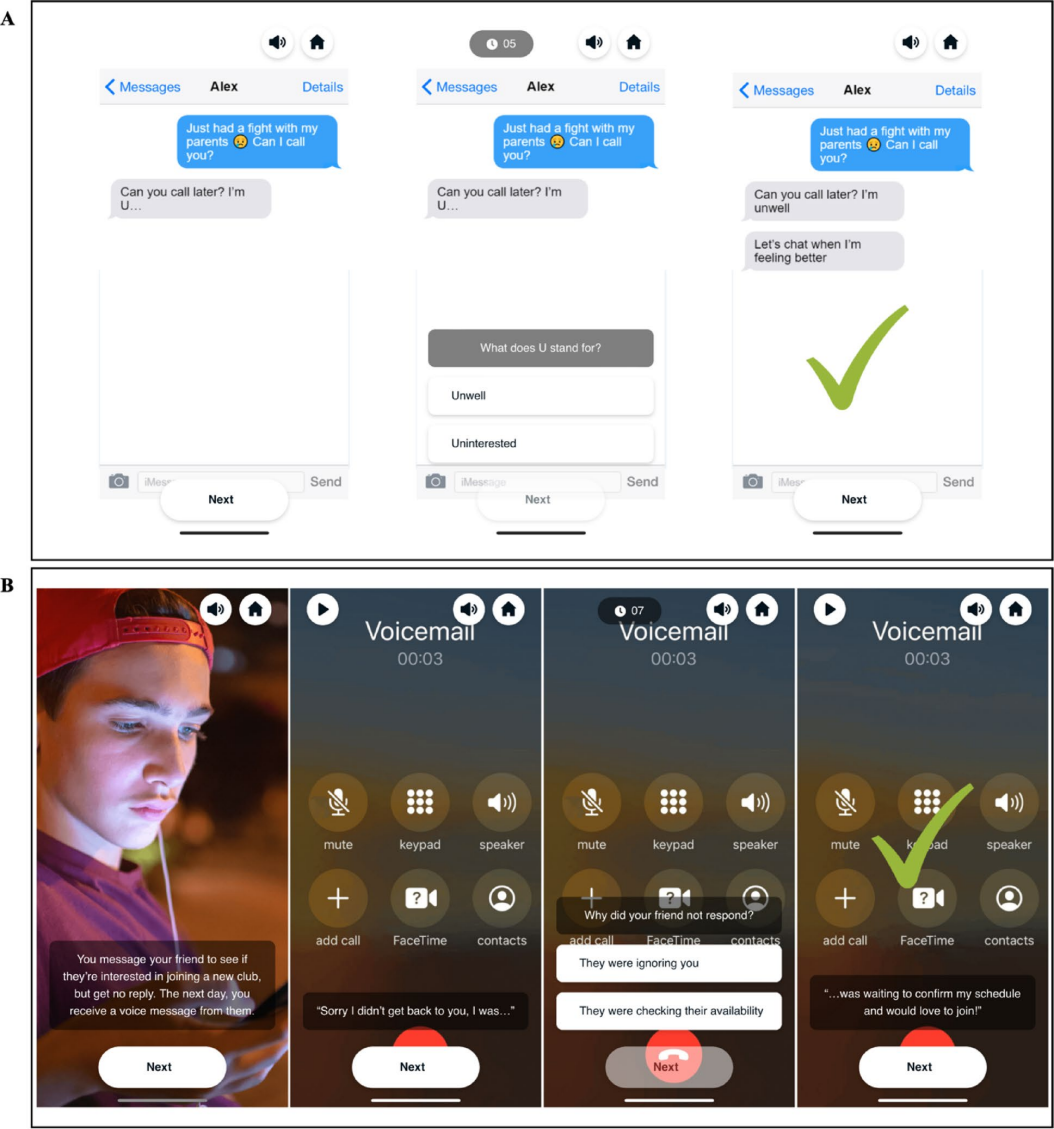

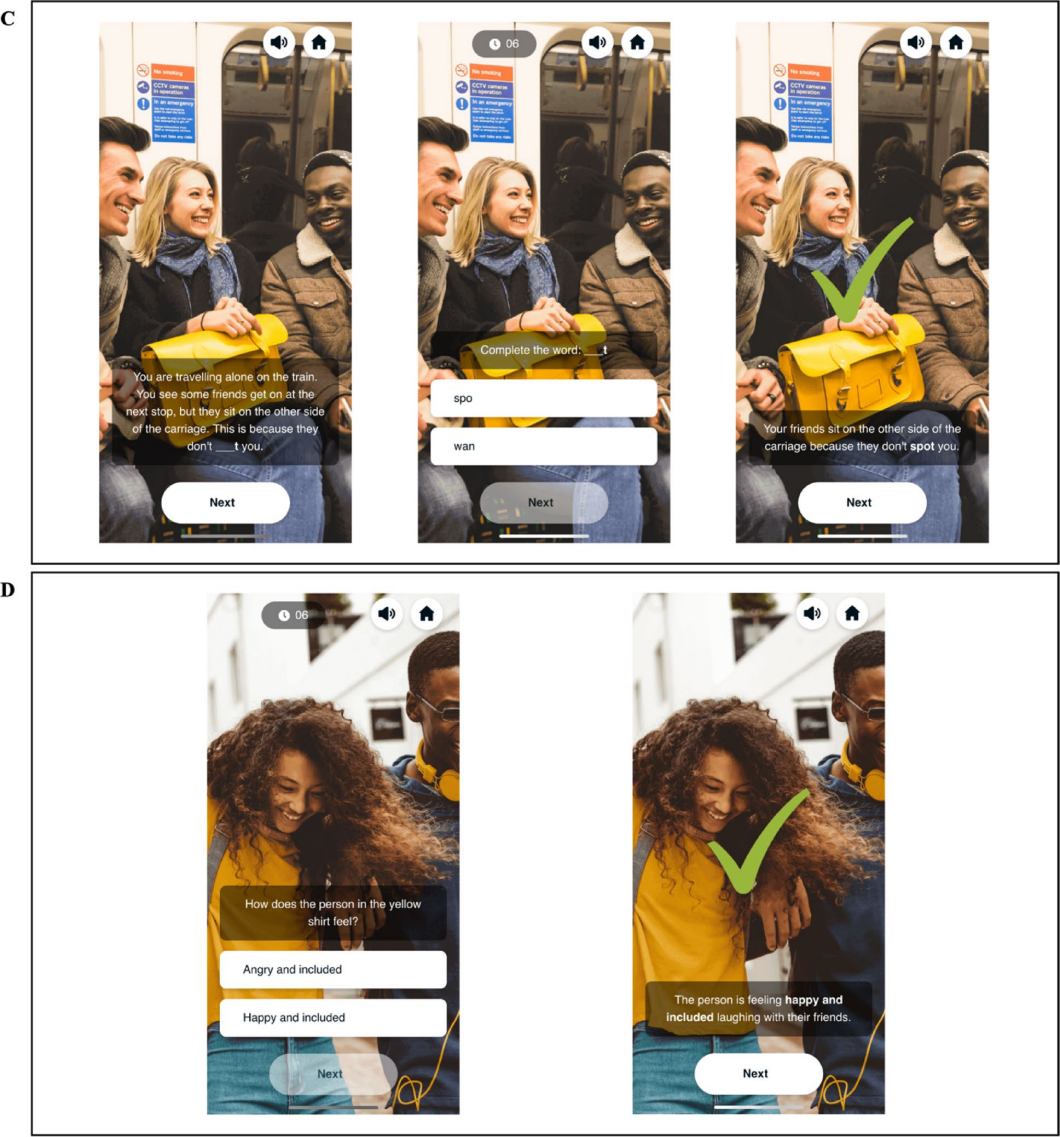
Depiction of the ambiguous social interaction scenarios *Note* Figure 3 depicts the four different ways the CBM-I scenarios were presented throughout training. **A** shows the text message format. Participants were first shown the text message interaction, then asked to choose between one of two options to resolve a missing fragment of the text message, before then being shown the scenario resolution and receiving feedback. **B** shows the audio format. Participants were first given a brief description of the context of the voicemail, then listened to the voicemail (they could also follow a transcript at the bottom of the screen), before being asked to choose between two options to resolve a missing fragment of the voicemail. On the final screen, the scenario was resolved and participants received feedback. **C** shows the narrative vignette format. Participants first read about the narrative ambiguous social interaction scenario, before being asked to complete a missing word fragment to resolve the scenario, and lastly being shown the scenario resolution and receiving feedback. **D** shows the emotion detection format. Participants were first asked to identify the emotional content of an image, before then being shown the scenario resolution and receiving feedback. In all four formats, a correct response was indicated by a green tick, while an incorrect response was indicated by a red cross.

**Cognitive interpretation bias modification**. The CBM-I component comprised seven ambiguous social interaction scenarios (trials) per training session. The ambiguous social interaction scenarios were presented in four interactive ways: (1) text message scenarios (Fig. 3A), a missing text fragment; (2) audio scenarios (Fig. 3B), a missing fragment from a voicemail recording; (3) narrative vignette scenarios (Fig. 3C), missing letters in a word fragment; and (4) emotion detection scenarios (Fig. 3D), where participants identified the emotional content of an image. Participants were required to resolve each scenario positively within 7 s, with feedback provided after each trial. Researchers have previously shown that adolescents can learn to positively resolve ambiguous social interaction scenarios [35].

**Fig. 4:**
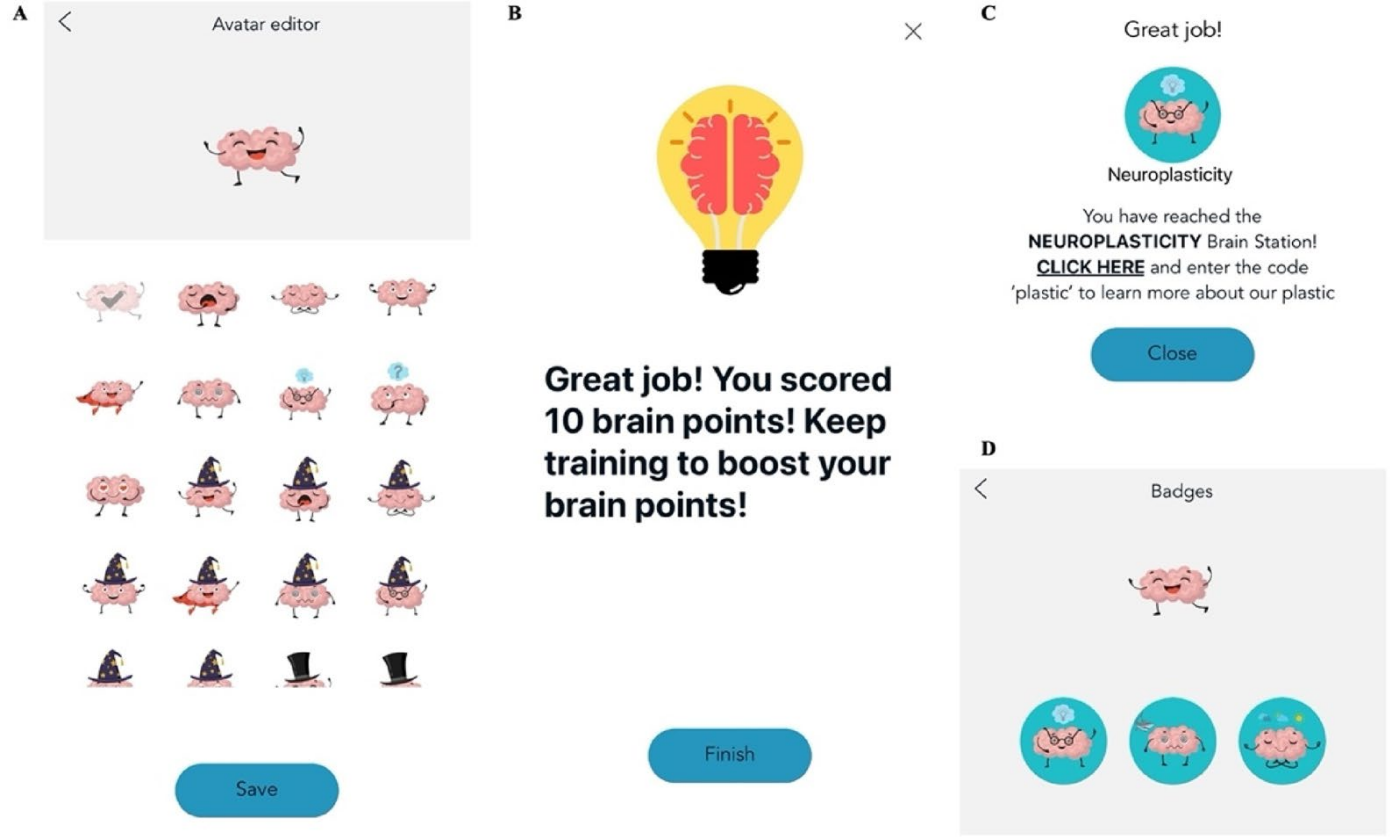
Depiction of gamification components *Note* Figure 4 depicts the gamification components of the SBT. **A** shows the avatars participants could choose after downloading the app. **B** shows an example of the “brain points” participants could receive for completing the affective control and CBM-I components of training. **C** depicts an example of the “brain stations” participants reached at the end of each training session. **D** shows some examples of the “brain badges” participants unlocked as they completed each training session.

**Gamification and incentivization.** To incentivise participation, several gamification components were built into the app. Participants could choose one of 45 brain avatars after downloading the app (Fig. 4A) and were awarded “brain points” based on their task performance throughout training (Fig. 4B).

During each training session participants also unlocked one fact about the human brain, and one fact about mental health (four facts on days 11 and 12). The 28 brain and mental health facts were designed to present psychoeducation content, including concepts such as neuroplasticity, in engaging text and video-based formats. Psychoeducation has been linked to increased treatment adherence and improved mental health outcomes [36, 37, 54].

Within each session, participants reached “brain stations” (Fig. 4C) as they progressed through the app, and after completing a training session in full they received “brain badges” (Fig. 4D). These stations and badges thematically matched the corresponding day’s brain/mental health facts and contained a code to unlock a linked webpage with additional resources related to that day’s brain/mental health topic (e.g., additional information on neuroplasticity). Once unlocked, badges remained accessible in a participant’s app profile for the duration of training.

#### Affective control training

Like the SBT, each AffeCT session started with 4 questions about mood, affect regulation, social context and current activity. AffeCT [55] then included the same dual *n*-back task as per the SBT. The only differences in the AffeCT dual *n*-back were that each block contained 20 + *n* trials, and each session was made up of 30 blocks. Participants were able to end each training session any time from 10 min onwards, and there was no limit on the number of training sessions participants could complete within a day. The first day of AffeCT training started at *n* = 1, with the level of *n* for each subsequent day of training starting at the average *n* achieved in the previous day’s training session.

### Procedure

All study components were completed online. After completing consent and assent procedures, participants completed all baseline assessments. They were then allocated to complete either AffeCT or SBT across two weeks, receiving an email from the research lab detailing how to download and access their assigned app to complete the training. Participants in both training groups received the same information about what training would involve prior to downloading their assigned training app. Throughout the two weeks of training, participants with notifications enabled received daily alerts from their training app to complete training, the first sent at 8am and the second at 5pm. Following the training, participants completed the post-training assessments (as baseline) and one-month follow-up assessment. All baseline, post-training and follow-up assessments were conducted using Gorilla Experiment Builder (https://gorilla.sc). Through the inbuilt participant messaging system within the Gorilla platform, participants were emailed links to complete the assessments at each time point. Those who did not complete the assessment at a given time point received two reminders to do so, the first sent 3 days after the initial email, and the second sent 7 days after the initial email. Participants were reimbursed with an AUD50 (GBP25) voucher for completing baseline, training and post-training assessments, and received an additional AUD10 (GBP5) voucher for completing the follow-up assessment. For a schematic overview of all measures and when they were administered, see Figure S2.

### Analyses

All statistical analyses were conducted using R version 4.2.1 [56]. Training groups (SBT vs. AffeCT) were compared on baseline characteristics and changes over time on the outcomes of interest prior to hypothesis testing. General linear models were conducted using the stats package [56]. Linear mixed models were conducted using the lme4 package [57], and their effect sizes were calculated using the MuMIn package [58]. Correlations were conducted using the apaTables package [59].

*Hypothesis 1 was tested with a general linear model, with training group entered as a predictor and minutes spent training as the outcome. Exploratory analyses were also run with app sessions and n-back usage separately added as the outcome in the model testing H1. App sessions were operationalised as the average number of times participants engaged in a training session in their assigned app, while n-back usage was operationalised as the average amount of time (minutes) participants spent on the n-back task of their assigned training app.*

Prior to testing hypotheses 2–3, exploratory analyses were run to investigate the effects of time (baseline vs. post-training) on mental health, interpretation bias, and affective control using mixed models. A Bonferroni correction of *p* *≤*.003 (0.05/16) was applied to account for 16 additional comparisons. Hypotheses 2 and 3 were tested using linear mixed models, with time (baseline vs. post-training) as a fixed within-subjects factor and Participant ID as a random effect. For models that included training group (SBT vs. AffeCT), this was added as a fixed between-subjects factor. As recommended for mixed effects models, effect sizes were calculated using r squared [60].

App usage (mins) was added as a predictor of affective control (H2a) and interpretation bias (H2b). To investigate H2c, training group (SBT vs. AffeCT) was added as a predictor to the model testing H2b.

*Hypothesis 3a was tested with training group (SBT vs. AffeCT) and baseline to post-training change in affective control as predictors of emotion regulation (as measured by the reappraisal subscale of the ERQ-CA and by the RTQ, entered into separate analyses). To test H3b, baseline to post-training changes in affective control and interpretation bias were separately added along with training group (SBT vs. AffeCT) as predictors of depression and anxiety respectively.*

To investigate the pre-registered exploratory analyses, assessing whether social sensitivity and concern for social risk would moderate improvements in affective control, social sensitivity and concern for social risk were separately added as fixed effects to the models testing H3 (first just as an interaction with time (baseline vs. post-training), then as an interaction with time (baseline vs. post-training) and training group (SBT vs. AffeCT).

### Data and code availability

On publication of the manuscript, de-identified data, syntax, and code supporting the conclusions of this article will be made available at the Open Science Framework (https://osf.io/preprints/psyarxiv/rpvh9). Study materials will not be made available, as most of the included images are licensed to the authors.

## Results

### Randomisation checks

Participants were randomly assigned to one of two training groups, SBT or AffeCT. Analyses of variance (ANOVA; continuous variables) and chi-squared tests (categorical variables) showed no group differences in baseline characteristics (see supplementary materials), except for self-rated emotion regulation (*F*(1140) = 4.33, *p* =.039), which was lower at baseline in the SBT compared to the AffeCT group.

### App acceptability

There were no significant differences in ratings of app helpfulness (*F*(1, 96) = 0.02, *p* =.896), ease of use (*F*(1, 96) = 0.18, *p* =.669), or likeability (*F*(1, 96) = 0.91, *p* =.342) between the training apps (Table 2).

**Table 2:** App acceptability variables means and standard deviations

	SBT mean (SD)	AffeCT mean (SD)
App helpfulness	2.07 (1.32)	2.10 (1.21)
App ease of use	2.95 (1.14)	2.85 (1.20)
App likeability	2.12 (1.27)	1.87 (1.22)

*App helpfulness indicates * how helpful participants found their assigned training app.* App ease of use* indicates how easy to use participants found their assigned training app. *App likeability* indicates how much participants liked using their assigned training app. All measures were rated from 0 (*not at all*) to 4 (*very*)

### App engagement

Means and standard deviations of training variables are presented in Table 3. A chi-squared test indicated that participants who were assigned to the SBT (*n* = 48 began training) app were significantly more likely to begin training than participants assigned to the AffeCT (*n* = 24 began training) app (*χ*^*2*^(1, *N* = 144) = 9.12, *p* =.003). Additionally, an independent samples *t*-test indicated that of those who began training, SBT (*M* = 6.61; *SD* = 7.27) participants engaged with significantly more training sessions on average than AffeCT (*M* = 3.61; *SD* = 7.08) participants (*t*(286) = −3.53, *p* <.001, 95% CI [−4.67, −1.33]).

**Fig. 5:**
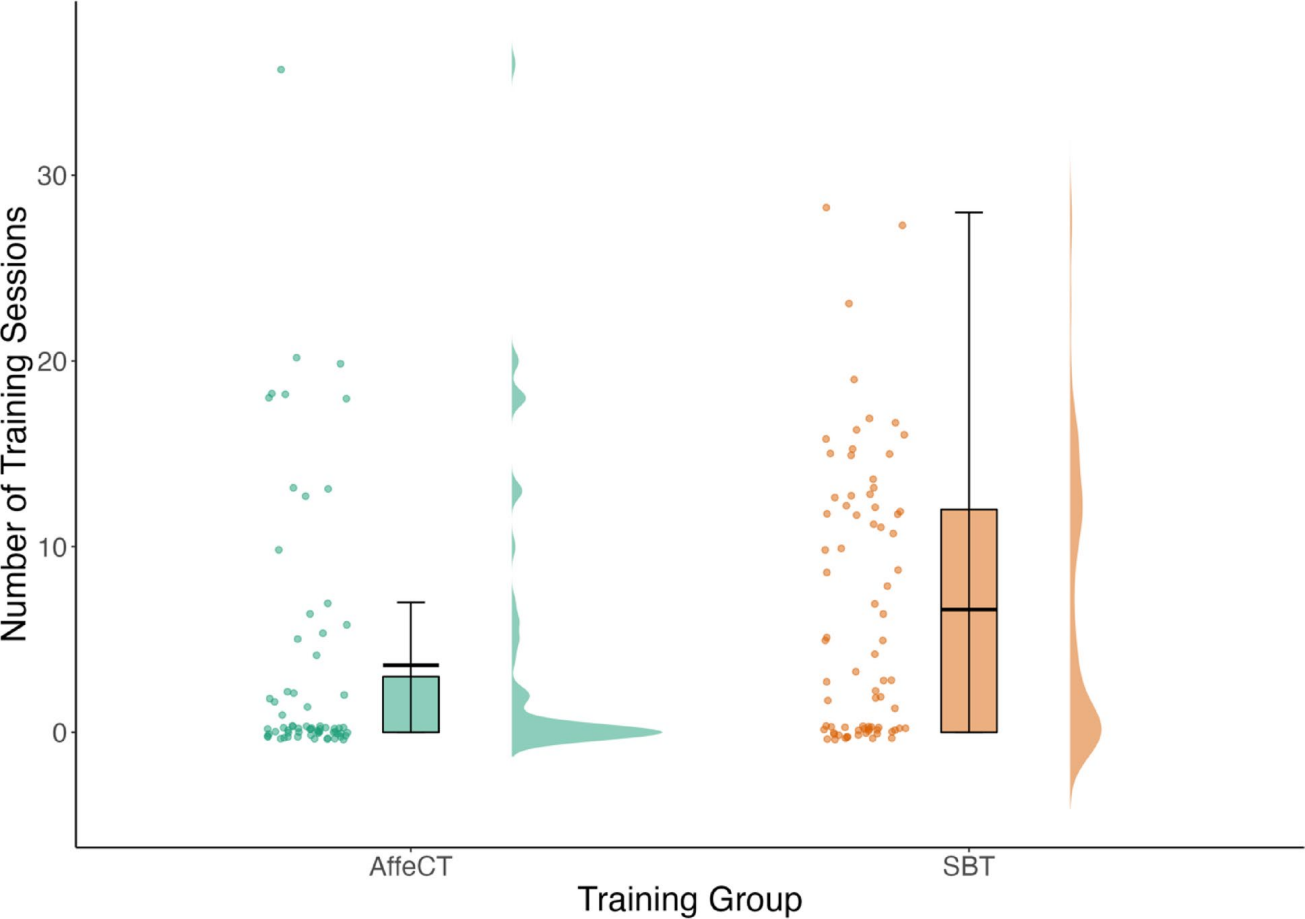
Number of training sessions engaged with. *Note* Effect of training group (SBT vs. AffeCT) on average number of sessions participants engaged with on their assigned training app.

Contrary to our hypothesis (H1), however, we did not observe a significant difference in amount of time (in minutes) spent training on the SBT compared to the AffeCT (*R*^*2*^ = 0.01, *F*_(1, 142)_ = 2.36, *p* =.127). Similarly, there was no significant difference in amount of time (in minutes) spent completing affective control training in the SBT compared to the AffeCT (*R*^*2*^ = −0.00, *F*_(1, 142)_ = 0.85, *p* =.357). However, exploratory analyses indicated a significant difference between number of training sessions participants engaged with in the SBT compared to the AffeCT (*R*^*2*^ = 0.04, *F*_(1, 142)_ = 6.20, *p* =.014), with the SBT group engaging in more training sessions (Fig. 5).

**Table 3:** Means and standard deviations of training variables across training groups

Variable	SBT (*n* = 77) mean (SD)	AffeCT (*n* = 67) mean (SD)
App usage	59.73 (67.24)	40.58 (81.84)
App sessions	6.61 (7.27)	3.61 (7.08)
N-back usage	82.37 (51.54)	110.62 (101.80)
Max N	3.81 (2.07)	4.29 (2.87)
Mean N	2.31 (1.23)	2.42 (1.32)
CBMI usage	44.31 (50.90)	N/A
CBMI RT	3889.267 (654.79)	N/A
CBMI accuracy	51.47(35.54)	N/A
Psychoed usage	20.51 (24.70)	N/A

*App usage* indicates average time (mins) spent in the assigned training app in total. *App sessions* indicates average number of times participants engaged in a training session in the assigned training app in total (a new engagement was counted each time there was a break longer than 5 mins between the end of the previous engagement and the start of the next engagement). *N-back usage* indicates amount of time (mins) spent training on the n-back task of the assigned training app. *Max N* indicates maximum N level reached across all trials of the assigned training app. *Mean N* indicates average N level reached across all trials of the assigned training app. *CBMI usage* indicates total time (mins) spent on CBMI tasks in the SBT app across all SBT app sessions. *CBMI RT* indicates average RT (ms) on correct CBMI trials across all SBT app sessions. *CBMI accuracy* indicates total correct CBMI trials across all SBT app sessions. *Psychoed usage* indicates total time (mins) spent on psychoeducation components in the SBT app across all SBT app sessions

### Training-related changes in affective control and interpretation bias

Means and standard deviations of outcome measures are presented in Table 4, and a breakdown by training group is presented in table S11. Across all participants, interpretation bias was reduced from baseline to post-training (*R*^*2*^*m* = 0.02, *R*^*2*^*c* = 0.70, *F* = 15.51, *df* = 115.71, *p* <.001). While affective control defined as the difference in RT for correct trials on the affective versus neutral condition of the 2-back task did not significantly differ from baseline to post-training (*R*^*2*^*m* = 0.00, *R*^*2*^*c* = 0. 38, *F* = 0.63, *df* = 126.19, *p* =.429), participants did get better at the task across conditions, as shown by a significant effect of time on RT on the 2-back task (*R*^*2*^*m* = 0.03, *R*^*2*^*c* = 0.64, *F* = 43.95, *df* = 383.00, *p* <.001). This indicates that when looking at RT generally, participants showed significant improvements in affective control from baseline to post-training.

However, in contrast with hypotheses H2a/b, time spent training did not interact with time (baseline vs. post-training) to predict improvements in affective control (*p* =.361; Table S1) or interpretation bias (*p* =.142; Table S2), though time spent training was significantly associated with improvements in interpretation bias after controlling for time point (*p* =.023; Table S2). Furthermore, there was no interaction between training group (SBT vs. AffeCT), time (baseline vs. post-training), and training time on interpretation bias (H2c; *p* =.106; Table S3).

The exploratory analysis including RT on correct trials of the untrained 2-back task (across the affective and neutral conditions) as outcome showed a significant improvement across time and greater improvement with more training time (Table S4). However there was no significant time x training time interaction.

**Table 4:** Means and standard deviations of outcome variables of interest across time points

Variable	Baseline mean (SD)	Post-training mean (SD)	Follow-up mean (SD)
Affective Control	− 4.35 (235.95)	12.40 (199.09)	N/A
Interpretation Bias	0.30 (0.24)	0.21 (0.21)	N/A
Emotion Regulation	27.08 (6.13)	28.00 (6.91)	27.37 (6.95)
Rumination	29.93 (8.80)	27.58 (9.22)	26.38 (8.95)
Depression	6.65 (6.61)	5.14 (5.94)	5.73 (6.67)
Anxiety	6.44 (6.06)	4.76 (5.28)	5.29 (5.95)
Social Sensitivity	25.19 (9.95)	24.48 (11.16)	23.33 (9.20)
Social Risk Concern	41.18 (18.06)	39.28 (16.29)	39.46 (16.00)
Affective Control Change (baseline to post-training)	N/A	− 20.13 (238.55)	N/A
Interpretation Bias Change (baseline to post-training)	N/A	0.05 (0.18)	N/A

*Affective Control* indicates average RT on correct trials of the emotional 2-back task minus average RT on correct trials of the neutral 2-back task. *Interpretation Bias* indicates proportion of negative grammatically correct sentences in the Scrambled Sentences Task. *Emotion Regulation* indicates total score on ERQ reappraisal subscale. *Rumination* indicates total score on RTQ. *Depression* indicates total score on PHQ. *Anxiety* indicates total score on GAD. *Social Sensitivity* indicates total score on O^2^S^3^. *Social Risk Concern* indicates average score of HSRQ rating scales. *Affective Control Change* change in average RT on correct trials of the emotional minus neutral 2-back task from baseline to post-training. *Interpretation Bias Change*  indicates change in proportion of negative grammatically correct sentences in the SST from baseline to post-training

### Training-related changes in mental health and emotion regulation

Exploratory analyses investigating baseline to post-training and follow-up changes in mental health showed no significant changes in symptoms of depression (Tables S5C-6C) and anxiety (Tables S5D-6D). While there were no significant changes in reappraisal (Tables S5A-6A), participants’ rumination decreased significantly from baseline to follow-up (Table S6B; Figure S3). These effects were consistent across training groups (i.e., no significant training group x time interaction; Tables S8-9).

In contrast with our third hypotheses, changes in affective control were not associated with changes in emotion regulation (*p* =.972; Table S9), depression (*p* =.368) or anxiety (*p* =.631; Table S10). Similarly, changes in interpretation bias were not associated with changes in depression (*p* =.821) or anxiety (*p* =.936; Table S10). These non-significant associations made the pre-registered exploratory analyses obsolete, however for completeness the code for these analyses is available with the code of the included analyses.

## Discussion

In this pre-registered study, we aimed to investigate the merits of introducing gamification components, such as badges and points, to standard affective control training paradigms to improve training uptake and adherence in an adolescent sample. We hypothesised that gamification would increase adolescent engagement with cognitive control training in affective contexts (i.e., affective control training), thus leading to improved affective control and, in turn, emotion regulation. We also directly trained emotion regulation abilities by targeting interpretation biases, aiming to decrease these and, by extension, improve mental health symptoms in adolescents. The results showed no significant differences in minutes spent training across the gamified and non-gamified training. However, exploratory analyses showed participants were twice as likely to start training when assigned to SBT compared to AffeCT training, and that participants engaged more with the gamified SBT (more sessions) compared to the AffeCT training. While training time was not associated with baseline to post-training improvements in affective control, when looking at overall performance on the 2-back task (i.e., neutral and affective condition) there was a significant change from baseline to post-intervention and there was a significant effect of training time. Similarly, interpretation bias improved from baseline to post-intervention and was associated with time spent training. Moreover, while training was not associated with significant changes in depression or anxiety symptoms and reappraisal capacity, there was a significant baseline to post-intervention reduction in rumination, which was maintained at follow-up.

Gamification did not lead to increased training time, as there was no difference in the amount of training time between the SBT and AffeCT groups. However, participants assigned to the SBT did engage in significantly more sessions than those assigned to the AffeCT. This is in line with previous research findings that gamification increased engagement with training (for a review, see 28), as despite not increasing the total time spent on the app, participants did seem to return to the gamified app more frequently than those who were engaging with its non-gamified counterpart. Importantly, training engagement was entirely self-motivated in the present study, as participants did not receive any additional monetary rewards for completing more training.

It is unclear, however, which specific components of the SBT may have led participants to return to it more often than those completing the AffeCT training. The two training apps did not differ in participants’ ratings of helpfulness, ease of use, or even likeability. Overall, training uptake and retention in the present study was quite low across the two apps. Of the 67 participants assigned to AffeCT, only 24 participants began training, and similarly only 47 of the 77 participants assigned to SBT began training. Furthermore, out of the minimum 12 sessions required for successful training completion across the two apps, participants only begun an average of 4 sessions in the AffeCT app and an average of 7 sessions in the SBT app. These differences are promising for the hypothesis that gamification may increase training engagement, as they indicate that participants were almost twice as likely to begin training and engaged on average with approximately twice as many sessions of training when assigned to the gamified SBT compared to the non-gamified AffeCT training. However, these findings are also in line with previous findings that cognitive training uptake in adolescents is quite low [24, 25], suggesting that the gamified factors included in the present study may still not be enough to fully incentivise participants to complete cognitive control training. Future studies aiming to incorporate gamification components into training programs should explicitly assess which components participants find most engaging.

 Interpretation bias was reduced from baseline to post-training, and while affective control measured as the difference between affective and neutral trials did not improve with training, affective and cognitive control as measured with overall performance on the untrained 2-back task also improved from baseline to post-intervention. Additionally, we observed significant effects of training time on interpretation bias and general performance of cognitive and affective control across baseline and post-training. These findings suggest that despite the low training uptake, engaging with the training apps led to overall improvements in interpretation bias and affective and cognitive control performance (i.e., combined performance across neutral and affective trials) from baseline to post-training.

This is in line with existing literature on cognitive control training studies, as researchers have previously found that cognitive training improves affective control (e.g., 33) and cognitive control (e.g., 24). The *n*-back task used to train cognitive control across both training groups in the present study was modelled after a similar n-back task that has previously been shown to significantly improve affective control [55]. Similarly, CBM-I training has been shown to successfully reduce interpretation bias [61–63], and the present task was modelled after a previously validated task [35]. Affective control training has also been associated with emotion regulation improvements [23], which may underly interpretation bias [64].

Interestingly, we also observed overall reductions in rumination from baseline that persisted over a one-month follow-up period. It has been hypothesised that reduced cognitive control may underly ruminative tendencies, with researchers finding associations between increased rumination and reduced cognitive control [65, 66]. However, previous studies employing 6 sessions of dual *n*-back training over a one week period, with the hope of increasing cognitive control and decreasing rumination as with the present study, did not find associations between training and cognitive improvements or differential effects of training on rumination [67]. The researchers proposed that this may have been due to insufficient training time, as they did observe a relationship between increased training time and decreased depressive symptomatology, which is closely associated with rumination [68], over time [67]. Indeed, in a study where participants completed 27 sessions of training involving multiple tasks, including a dual *n*-back task, over a period of 4 weeks, researchers observed improvements in negative mood in the cognitive control training group [69], a factor that is closely linked to rumination [70, 71]. Other studies have similarly found that cognitive training decreased ruminative tendencies (e.g., 72,73). Together, these findings suggest that cognitive training can successfully reduce rumination over time.

The findings presented here should be considered within the context of the study’s limitations. First, while the power calculation we conducted prior to participant recruitment assumed a non-compliance rate of 40%, training compliance rates were even lower in the present sample (51% across the whole sample), especially for participants assigned to the AffeCT training group (64%). As a result, there may have been insufficient power to detect training effects in the present sample. Second, as the study’s aim was to demonstrate increased engagement with a training paradigm through gamification, there was no control group that completed non-affective control training. That is, observed effects could be due to placebo effects of engaging in cognitive training. However, if the observed effects are genuine, it suggests that even a limited amount of training can improve affective and cognitive control, interpretation bias, and rumination in adolescents. Future studies should seek to replicate these effects including a placebo-training group, thus conducting efficacy trials to investigate the effectiveness of such training programs as preventive interventions.

A further limitation is the study’s follow-up period. Design and production of the SBT app began in early 2020. However, production of the app faced significant delays throughout the COVID-19 pandemic, and participant recruitment did not begin until mid-2023. Due to limitations with the grant expiry, the follow-up time point had to be changed from six-months to one-month post-training. Additionally, while recruitment and baseline assessments were originally proposed to be conducted in-person, the study had to be moved fully online and relied on social media advertisements to complete recruitment within the limited time left to conduct the study. While online recruitment benefitted the samples’ representativeness [74], it required the detection of fraudulent participants. The current study implemented a range of procedures to identify fraudulent participants that can be adopted in future research.

## Conclusion

Despite its limitations, this study provides preliminary evidence that gamification may be a viable tool for encouraging adolescents to begin and continue engaging with cognitive training. The results provide further tentative support for even limited amounts of affective control training’s potential to improve affective and cognitive control and reduce interpretation bias and rumination. If gamification effects can be further maximised to increase training adherence to apps such as the SBT, these apps have the potential to then be further developed as preventive interventions for adolescent mental health disorders and disseminated at larger scales, as the training is conducted online and at no cost to users. Research has shown that across 21 different countries, 90% of individuals under 24 years have access to the internet [75]. Such interventions would therefore be easily accessible to youth worldwide, making them promising tools for targeting the leading cause of disability in adolescents.

## Electronic supplementary material

Supplementary Material 1

## Data Availability

On publication of the manuscript, de-identified data and code necessary to reproduce the analyses presented here will be made publicly accessible at the following URL: https://osf.io/preprints/psyarxiv/rpvh9. The materials necessary to attempt to replicate the findings presented here are not publicly available, as most of the included images are licensed to the authors.

## Abbreviations

AffeCT: Non-gamified Affective Control Training
ANOVA: Analysis of Variance
CBM-I: Cognitive Interpretation Bias Modification
CI: Confidence Interval
ERQ: Emotion Regulation Questionnaire– child and adolescent version
GAD: Generalized Anxiety Disorder Scale
HSRQ: Health and Social Risk Questionnaire
M: Mean
O^2^S^3^: Online and Offline Social Sensitivity Scale
PHQ: Patient Health Questionnaire - Adolescent
RT: Reaction Time
RTQ: Repetitive Thinking Questionnaire
SBT: Social Brain Train
SD: Standard Deviation
SES: socio-economic status
SST: Scrambled Sentences Task
