# Supplementary material for: Investigating the effects of a novel gamified cognitive training on adolescent mental health
Source: Child Adolesc Psychiatry Ment Health. 2025 Jul 3;19:72. doi: 10.1186/s13034-025-00917-1 (PMC12225106; doi:10.1186/s13034-025-00917-1)
Supplement: Supplementary file 1 — Supplementary Material 1 [file 13034_2025_917_MOESM1_ESM.docx]

**Investigating the effects of a novel gamified cognitive training on adolescent mental health**

**Supplementary Materials**

**Figure S1**

*Participant Recruitment, Inclusion and Exclusion*

*
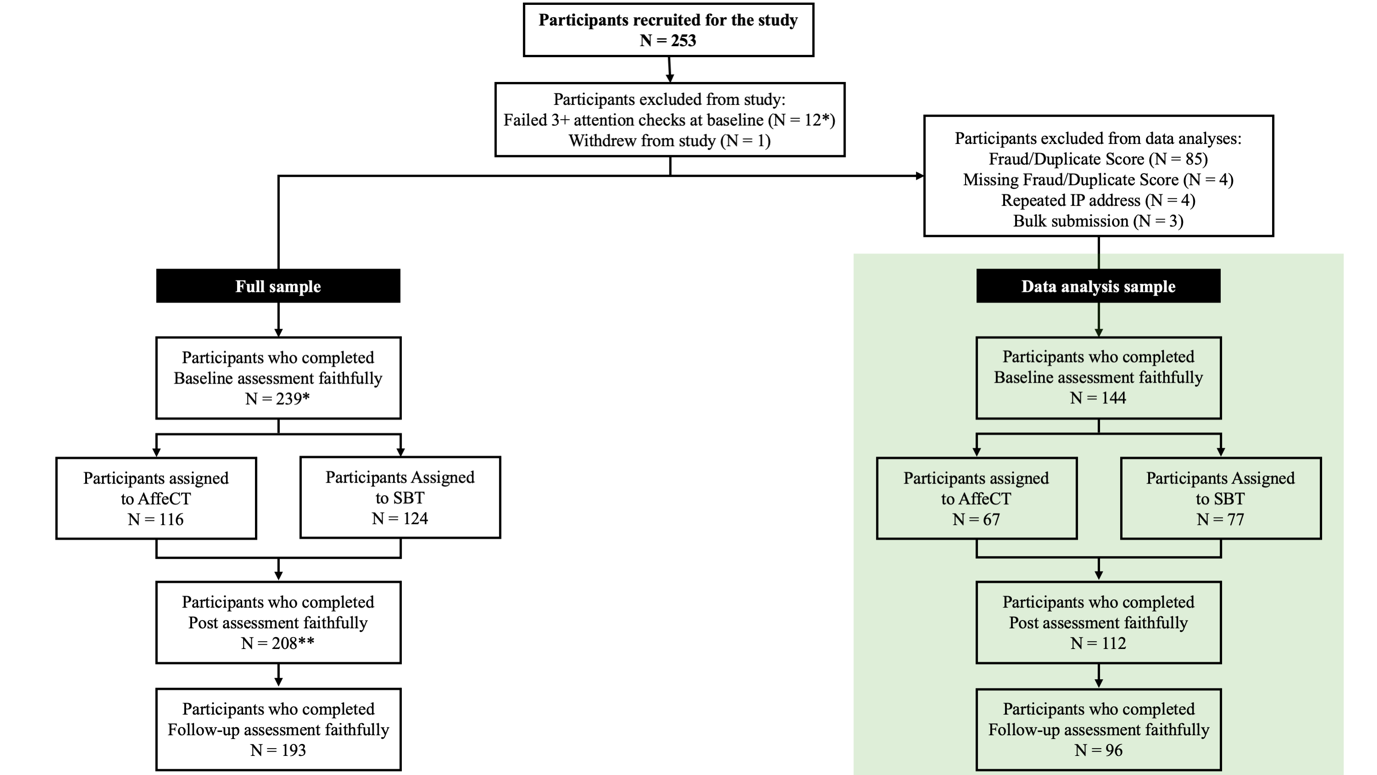
*

*Note*. *1 additional participant failed 3+ attention checks at baseline, but due to experimenter error was still assigned to a training group and completed training; this participant was therefore excluded from baseline but retained for training, post-training, and follow-up assessments in the full data sample. **4 participants completed post-training and follow-up measures at the same time; their questionnaire responses were therefore removed from the post-training time point, and the questionnaire responses they completed first were retained and used as follow-up questionnaire responses; their post-training task data was retained and we corrected for time between training completion and post-training task measure completion in the relevant analyses (H2-3); therefore, while *N* = 208 participants completed post-training task measures, only *N* = 204 completed post-training questionnaire measures.

**Figure S2**

*Study Procedure Flow Chart*

*
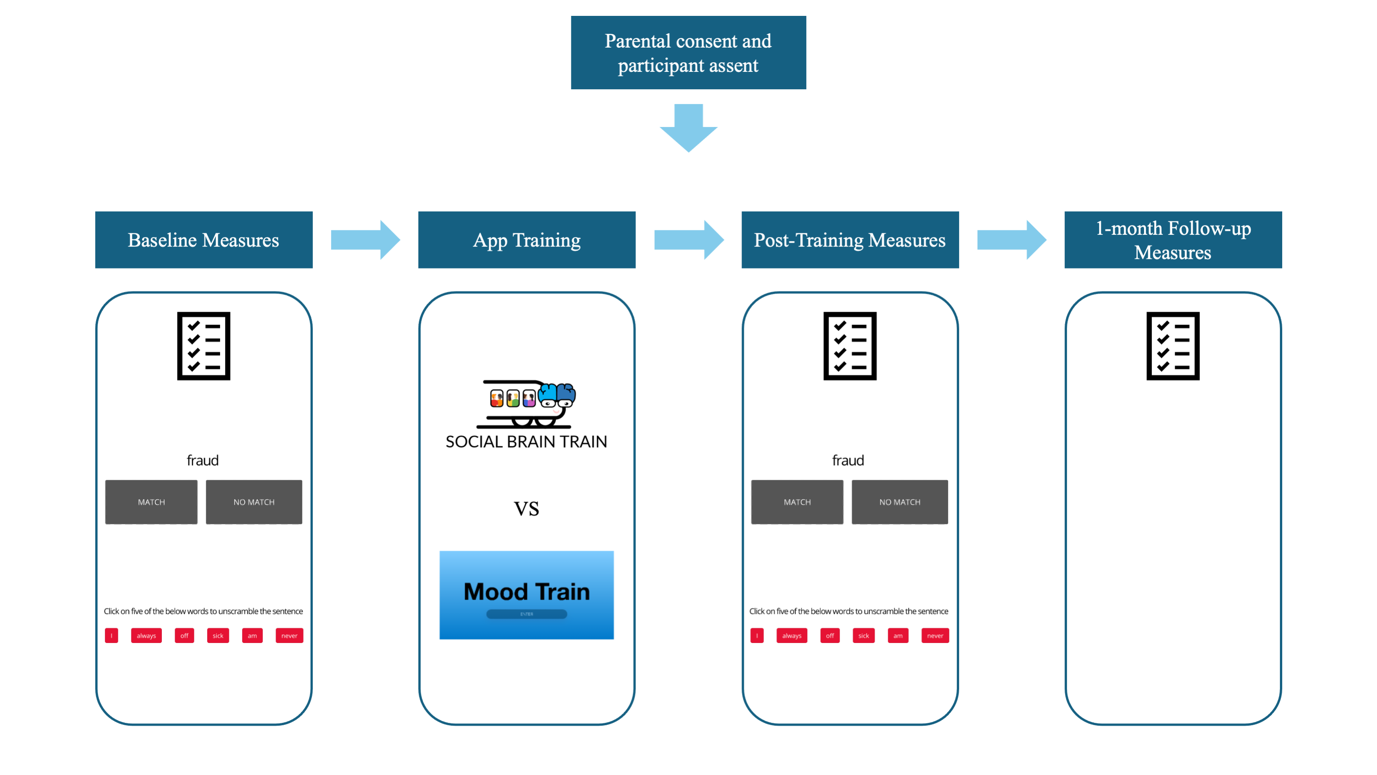
*

*Note*. The figure depicts the study procedure.
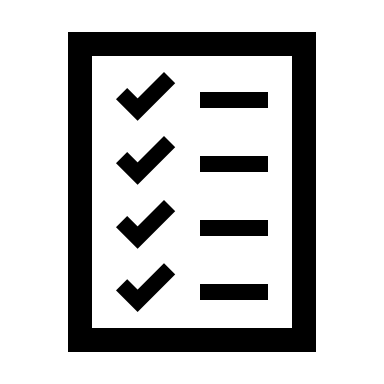
= self-report measures (demographics questionnaire (only at baseline), daily screentime usage, ERQ-CA, RTQ-10, PHQ-A, CALIS; GAD-7, O^2^S^3^; HSRQ, training app acceptability (only at follow-up)); = 2-back task (emotional and neutral conditions); = scrambled sentences task;
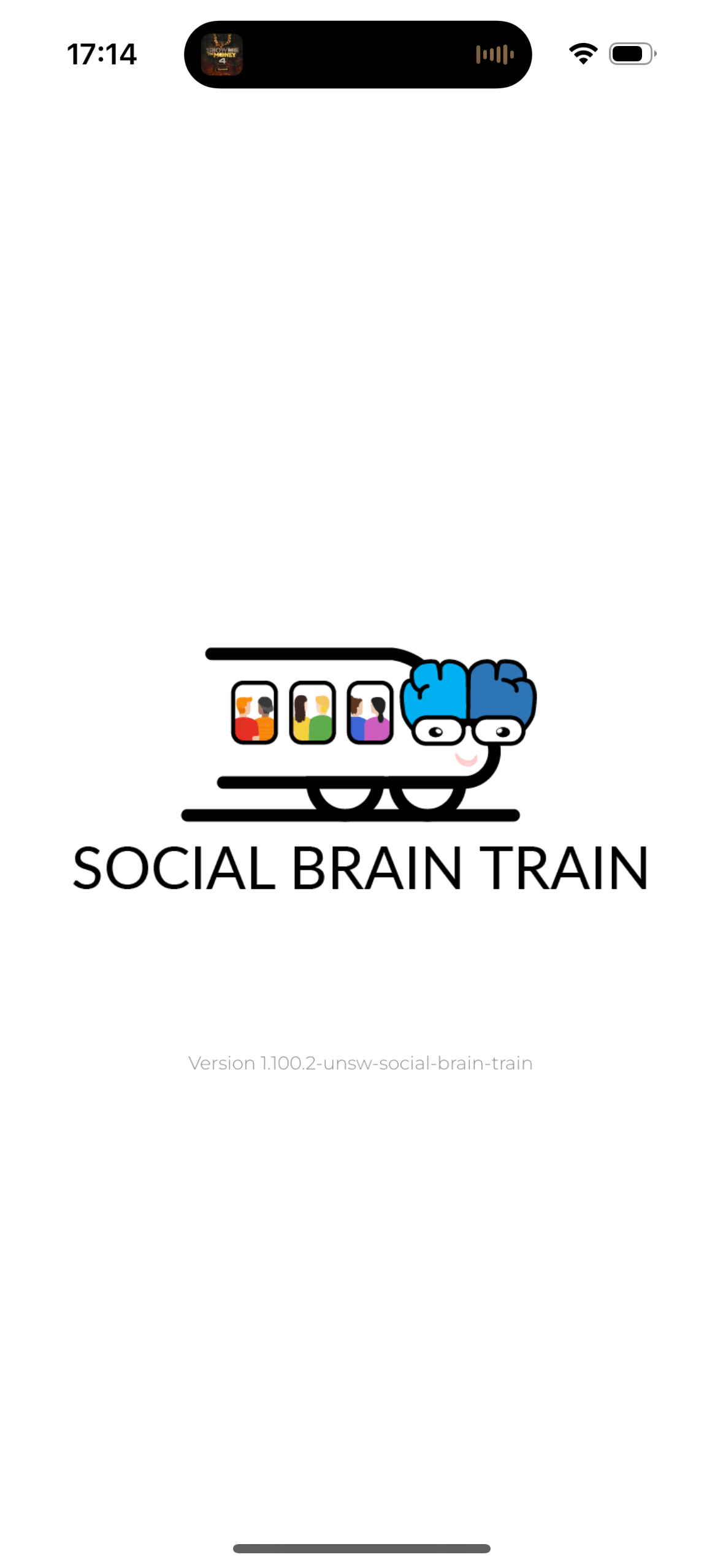
= Social Brain Train app training group;
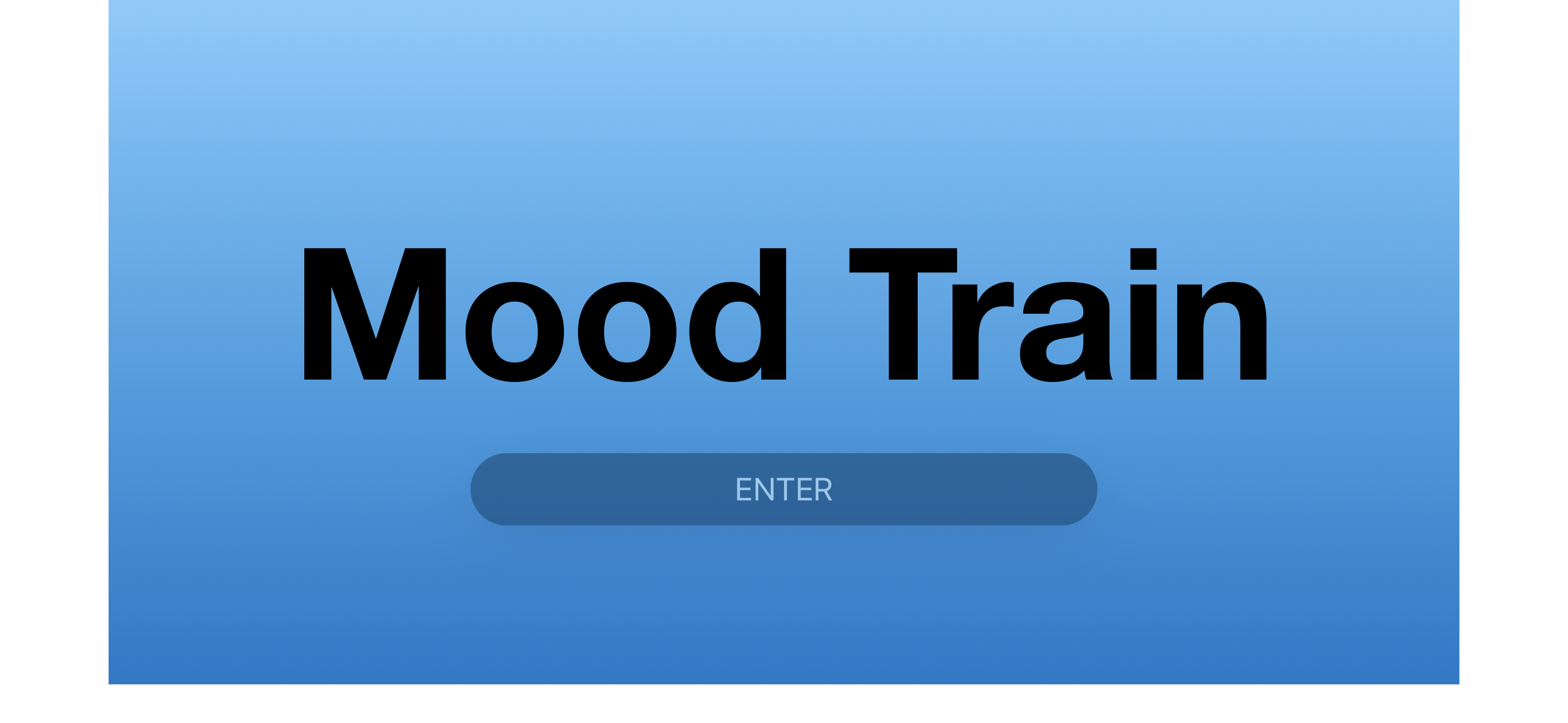
= AffeCT app training group.


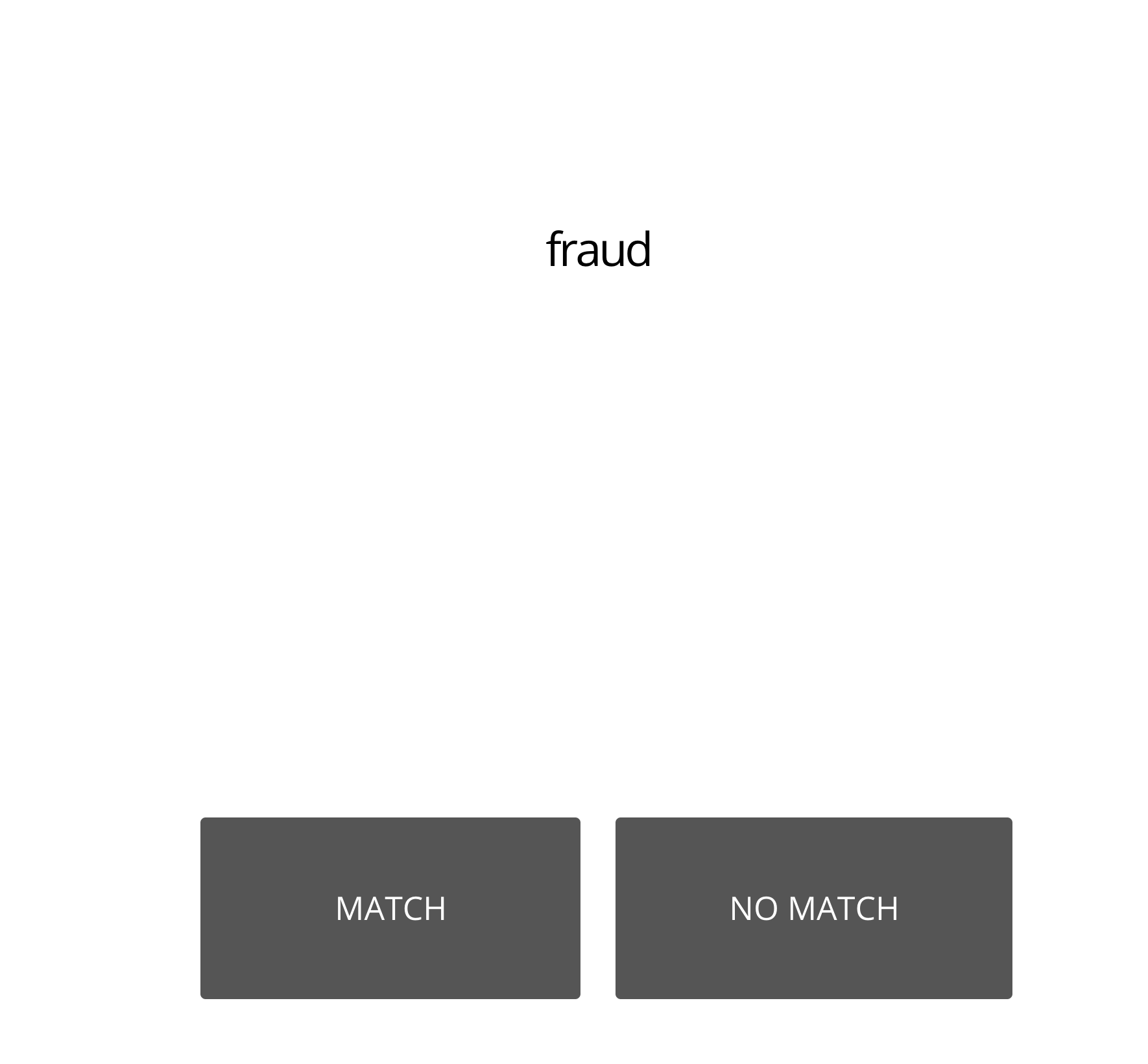

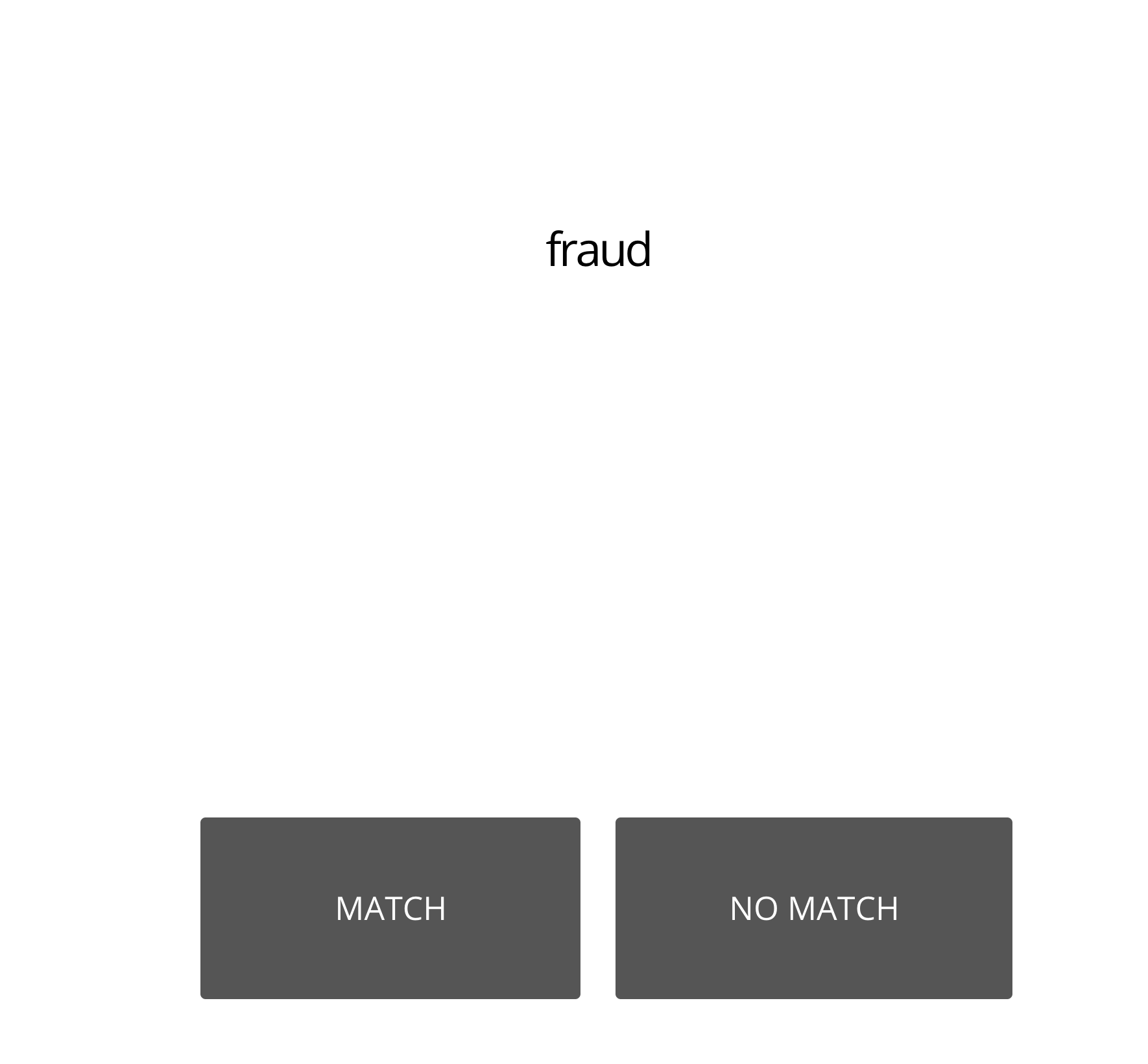

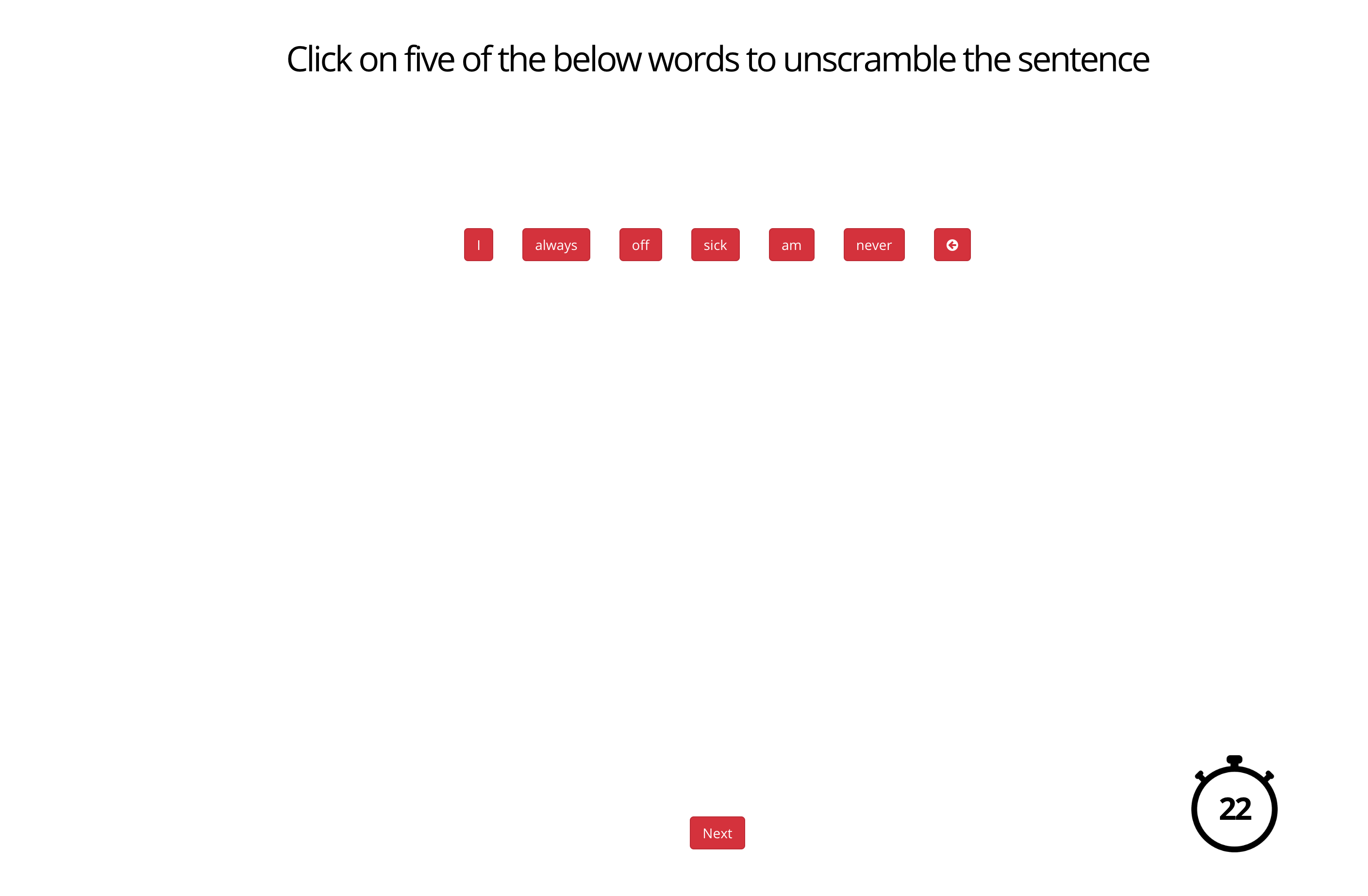

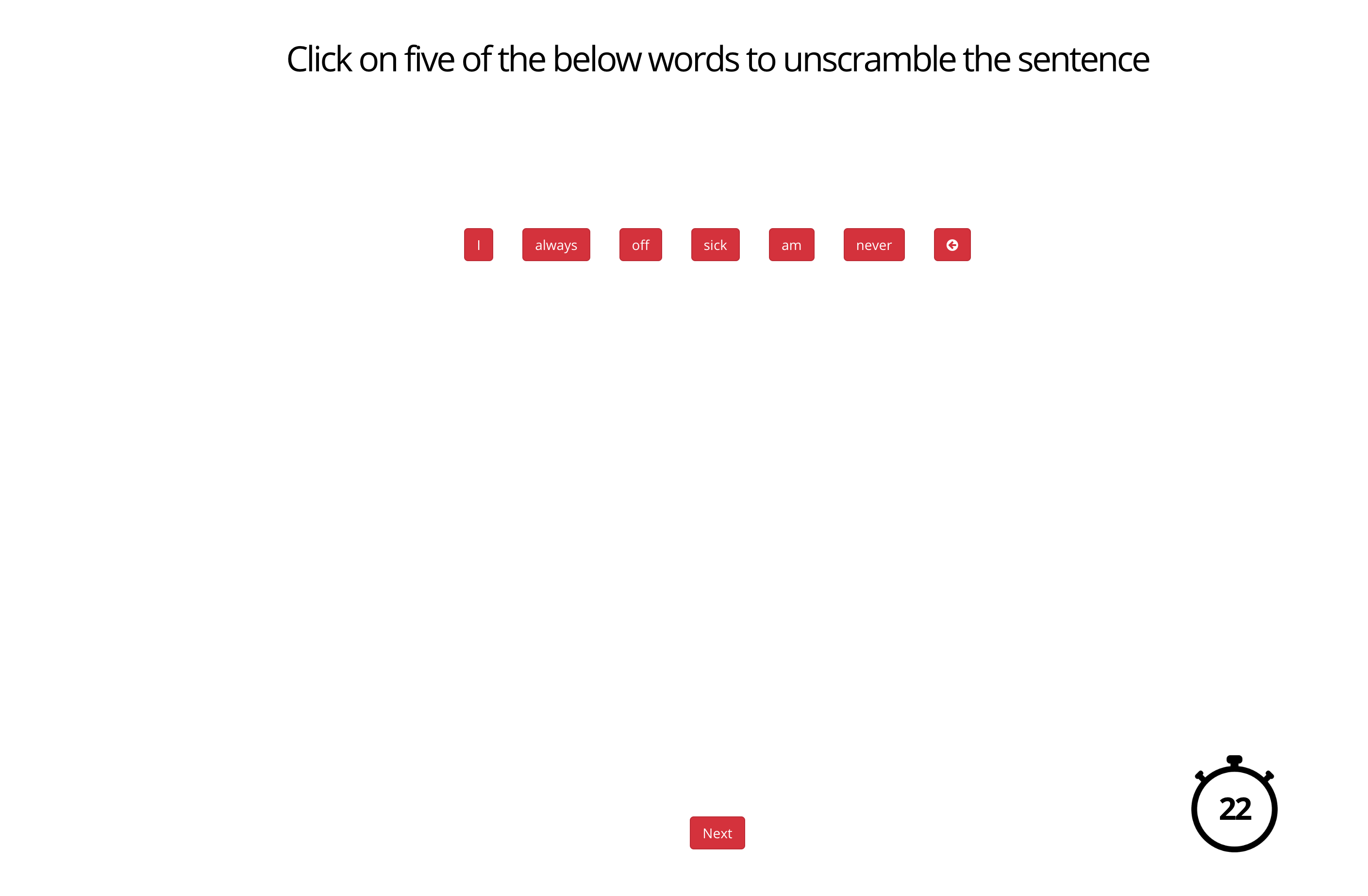


**Supplementary Results**

**Randomisation Checks**

There were no significant differences in participant age (*F*(1, 142) = 0.12, *p* = .727), gender (*χ ^2^*(2, *N* = 144) = 2.03, *p* = .361), country (*χ ^2^*(3, *N* = 144) = 2.05, *p* = .562), ethnicity (*χ ^2^*(6, *N* = 144) = 12.27, *p* = .056), or SES (*F*(1, 132) = 0.00, *p* = .970) across the different training groups. Similarly, there were no significant differences in baseline participant self-rated rumination (*F*(1, 140) = 0.48, *p* = .489), depressive symptoms (*F*(1, 141) = 0.49, *p* = .487), anxiety symptoms (*F*(1, 140) = 2.83, *p* = .095), social sensitivity (*F*(1, 121) = 3.85, *p* = .052), or social risk concern (*F*(1, 142) = 0.65, *p* = .421) across the training groups, indicating successful randomization of participants.

**Table S1**

*Summary of H2a Analyses*

|  | **Affective control** | | | |
| --- | --- | --- | --- | --- |
| *Predictors* | *df* | *F* | *p* | *R^2^m/R^2^c* |
|  |  |  |  | 0.02/0.32 |
| Time | 107.00 | 0.04 | .845 |  |
| App usage | 192.77 | 0.22 | .636 |  |
| Days since training | 106.00 | 0.48 | .488 |  |
| Time x App usage | 107.00 | 0.84 | .361 |  |

*Note*. Bolded text indicates statistically significant effects. **Time** indicates time point of measure completion (baseline vs post-training). **App usage** indicates average time (mins) spent in the assigned training app in total. **Days since training** indicates time between training completion and post task measure completion. **Affective control** was operationalised as average RT on correct trials of the emotional 2-back task minus average RT on correct trials of the neutral 2-back task (measured at baseline and post-training).

**Table S2**

*Summary of H2b Analyses*

|  | **Interpretation bias** | | | |
| --- | --- | --- | --- | --- |
| *Predictors* | *df* | *F* | *p* | *R^2^m/R^2^c* |
|  |  |  |  | 0.07/0.69 |
| Time | 105.83 | 3.21 | .076 |  |
| App usage | **145.60** | **5.25** | **.023** |  |
| Days since training | **105.89** | **4.03** | **.047** |  |
| Time x App usage | 105.30 | 2.19 | .142 |  |

*Note*. Bolded text indicates statistically significant effects. **Time** indicates time point of measure completion (baseline vs post-training). **App usage** indicates average time (mins) spent in the assigned training app in total. **Days since training** indicates time between training completion and post task measure completion. **Interpretation bias** was operationalised as the proportion of negative grammatically correct sentences in the Scrambled Sentences Task (measured at baseline and post-training).

**Table S3**

*Summary of H2c Analyses*

|  | **Interpretation bias** | | | |
| --- | --- | --- | --- | --- |
| *Predictors* | *df* | *F* | *p* | *R^2^m/R^2^c* |
|  |  |  |  | 0.13/0.72 |
| Time | 103.05 | 0.04 | .848 |  |
| App usage | 141.05 | 0.34 | .558 |  |
| Training group | **144.63** | **5.06** | **.026** |  |
| Days since training | **103.89** | **4.51** | **.036** |  |
| Time x App usage | 103.05 | 0.12 | .733 |  |
| Time x Training group | 103.99 | 1.67 | .199 |  |
| App usage x Training group | 142.86 | 1.07 | .303 |  |
| Time x App usage x Training group | 103.49 | 2.66 | .106 |  |

*Note*. Bolded text indicates statistically significant effects. **Time** indicates time point of measure completion (baseline vs post-training). **App usage** (mins) indicates average time (mins) spent in the assigned training app in total. **Training group** indicates app training group (SBT vs AffeCT). **Days since training** indicates time between training completion and post task measure completion. **Interpretation bias** was operationalised as the proportion of negative grammatically correct sentences in the Scrambled Sentences Task (measured at baseline and post-training).

**Table S4**

*Summary of exploratory H2a 2-back performance analysis*

|  | **2-back RT** | | | |
| --- | --- | --- | --- | --- |
| *Predictors* | *df* | *F* | *p* | *R^2^m/R^2^c* |
|  |  |  |  | 0.18/0.70 |
| Time | **325.00** | **21.76** | **<.001** |  |
| App usage | **133.42** | **24.19** | **<.001** |  |
| Days since training | **106.00** | **6.61** | **.012** |  |
| Time x App usage | 325.00 | 0.49 | .485 |  |

*Note*. Bolded text indicates statistically significant effects. **Time** indicates time point of measure completion (baseline vs post-training). **App usage** (mins) indicates average time (mins) spent in the assigned training app in total. **Days since training** indicates time between training completion and post task measure completion. **2-back RT** indicates reaction time on correct trials of the untrained 2-back task.

**Table S5**

*Exploratory effects of time (baseline vs post-training) on outcomes of interest*

| ***A:*** *Effect of time on emotion regulation* | | | | | | | | |
| --- | --- | --- | --- | --- | --- | --- | --- | --- |
|  | **Emotion Regulation** | | | | | | | |
| *Predictors* | *df* | | *F* | | *p* | | *R^2^m/R^2^c* | |
|  |  | |  | |  | | 0.00/0.65 | |
| Time | 114.84 | | 0.17 | | .685 | |  | |
| ***B:*** *Effect of time on rumination* | | | | | | | | |
|  | | **Rumination** | | | | | | |
| *Predictors* | | *df* | | *F* | | *p* | | *R^2^m/R^2^c* |
|  | |  | |  | |  | | 0.02/0.61 |
| Time | | 116.22 | | 9.06 | | .003 | |  |
| ***C:*** *Effect of time on depression* | | | | | | | | |
|  | | **Depression** | | | | | | |
| *Predictors* | | *df* | | *F* | | *p* | | *R^2^m/R^2^c* |
|  | |  | |  | |  | | 0.00/0.90 |
| Time | | 107.99 | | 0.42 | | .516 | |  |
| ***D:*** *Effect of time on anxiety* | | | | | | | | |
|  | | **Anxiety** | | | | | | |
| *Predictors* | | *df* | | *F* | | *p* | | *R^2^m/R^2^c* |
|  | |  | |  | |  | | 0.00/0.83 |
| Time | | 109.08 | | 0.97 | | .328 | |  |

*Note*. Bolded text indicates statistically significant effects (*p* < .003). **Time** indicates time point of measure completion (baseline vs post-training). **Emotion Regulation** was operationalised as total score on ERQ reappraisal subscale. **Rumination** was operationalised as total score on the RTQ. **Depression** was operationalised as total score on PHQ. **Anxiety** was operationalised as total score on GAD.

**Table S6**

*Exploratory effects of time (baseline vs post-training vs follow-up) on outcomes of interest*

| ***A:*** *Effect of time on emotion regulation* | | | | | | | | |
| --- | --- | --- | --- | --- | --- | --- | --- | --- |
|  | **Emotion Regulation** | | | | | | | |
| *Predictors* | *df* | | *F* | | *p* | | *R^2^m/R^2^c* | |
|  |  | |  | |  | | 0.00/0.61 | |
| Time | 211.20 | | 1.36 | | .259 | |  | |
| ***B:*** *Effect of time on rumination* | | | | | | | | |
|  | | **Rumination** | | | | | | |
| *Predictors* | | *df* | | *F* | | *p* | | *R^2^m/R^2^c* |
|  | |  | |  | |  | | 0.03/0.60 |
| Time | | **213.12** | | **12.81** | | **<.001** | |  |
| ***C:*** *Effect of time on depression* | | | | | | | | |
|  | | **Depression** | | | | | | |
| *Predictors* | | *df* | | *F* | | *p* | | *R^2^m/R^2^c* |
|  | |  | |  | |  | | 0.00/0.86 |
| Time | | 204.07 | | 0.42 | | .656 | |  |
| ***D:*** *Effect of time on anxiety* | | | | | | | | |
|  | | **Anxiety** | | | | | | |
| *Predictors* | | *df* | | *F* | | *p* | | *R^2^m/R^2^c* |
|  | |  | |  | |  | | 0.00/0.81 |
| Time | | 204.94 | | 0.48 | | .619 | |  |

*Note*. Bolded text indicates statistically significant effects (*p* < .003). **Time** indicates time point of measure completion (baseline vs post-training vs follow-up). **Emotion Regulation** was operationalised as total score on ERQ reappraisal subscale. **Rumination** was operationalised as total score on the RTQ. **Depression** was operationalised as total score on PHQ. **Anxiety** was operationalised as total score on GAD.

**Figure S3**

*Exploratory effect of time (baseline vs post-training vs follow-up) on rumination*

**
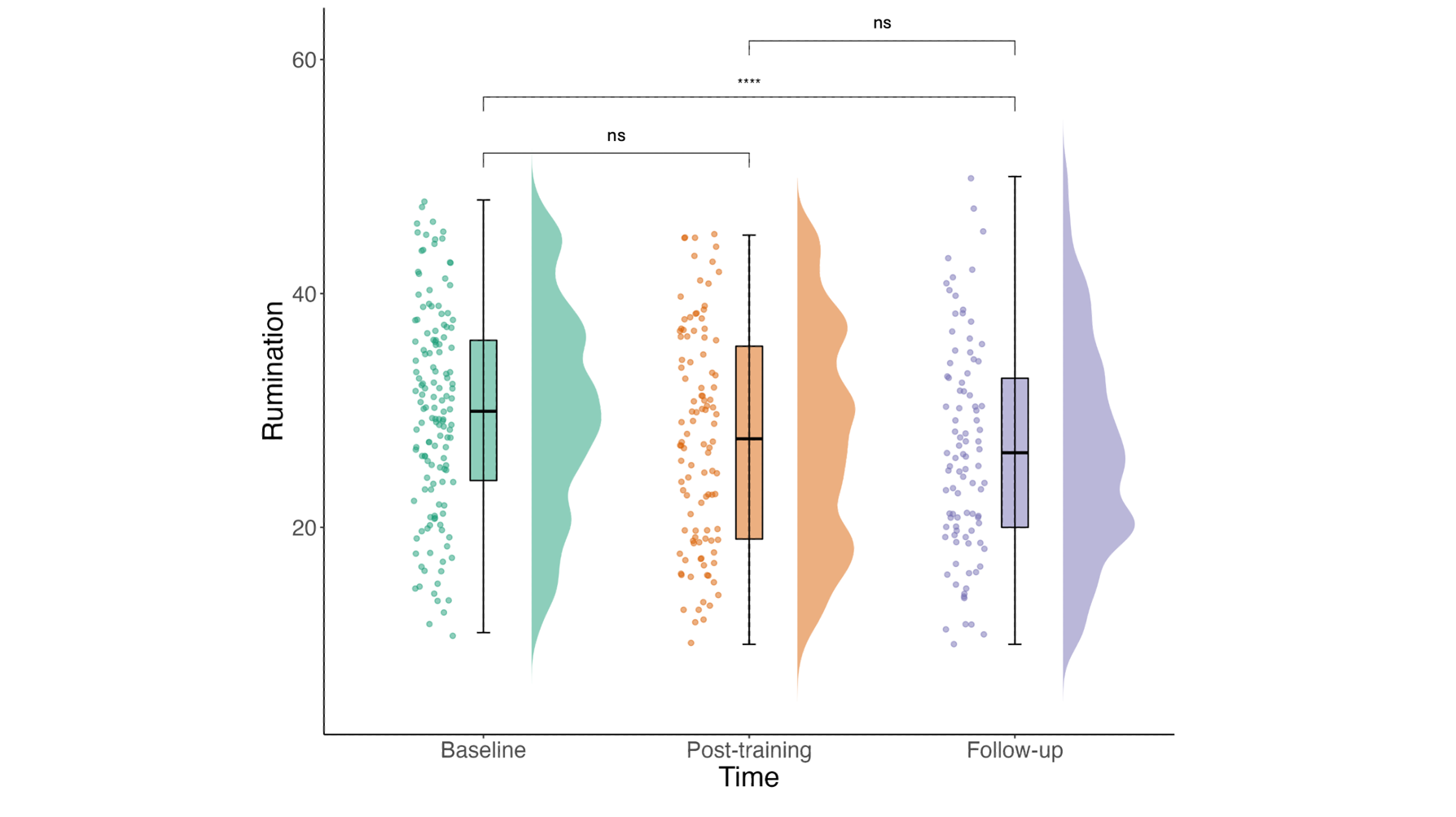
**

*Note*. Effect of time (baseline vs pos-training vs follow-up) on self-rated rumination. Rumination was operationalised as total score on the RTQ.

**Table S7**

*Exploratory effects of time (baseline vs post-training) and training group (SBT vs AffeCT) on outcomes of interest*

| ***A:*** *Effect of time and training group on emotion regulation* | | | | |
| --- | --- | --- | --- | --- |
|  | **Emotion Regulation** | | | |
| *Predictors* | *df* | *F* | *p* | *R^2^m/R^2^c* |
|  |  |  |  | 0.02/0.66 |
| Time | 115.94 | 1.42 | .236 |  |
| Training group | 184.79 | 3.77 | .054 |  |
| Time x Training group | 114.03 | 3.84 | .053 |  |
| ***B:*** *Effect of time and training group on rumination* | | | | |
|  | **Rumination** | | | |
| *Predictors* | *df* | *F* | *p* | *R^2^m/R^2^c* |
|  |  |  |  | 0.02/0.61 |
| Time | 117.94 | 4.48 | .036 |  |
| Training group | 191.85 | 0.31 | .578 |  |
| Time x Training group | 115.73 | 0.03 | .860 |  |
| ***C:*** *Effect of time and training group on depression* | | | | |
|  | **Depression** | | | |
| *Predictors* | *df* | *F* | *p* | *R^2^m/R^2^c* |
|  |  |  |  | 0.01/0.90 |
| Time | 107.22 | 2.45 | .121 |  |
| Training group | 153.46 | 0.61 | .435 |  |
| Time x Training group | 106.97 | 2.26 | .135 |  |
| ***D:*** *Effect of time and training group on anxiety* | | | | |
|  | **Anxiety** | | | |
| *Predictors* | *df* | *F* | *p* | *R^2^m/R^2^c* |
|  |  |  |  | 0.01/0.83 |
| Time | 108.86 | 0.01 | .914 |  |
| Training group | 161.72 | 2.44 | .120 |  |
| Time x Training group | 108.18 | 0.56 | .455 |  |

*Note*. Bolded text indicates statistically significant effects (*p* < .003). **Time** indicates time point of measure completion (baseline vs post-training). **Emotion Regulation** was operationalised as total score on ERQ reappraisal subscale. **Rumination** was operationalised as total score on the RTQ. **Depression** was operationalised as total score on PHQ. **Anxiety** was operationalised as total score on GAD.

**Table S8**

*Exploratory effects of time (baseline vs post-training vs follow-up) and training group (SBT vs AffeCT) on outcomes of interest*

| ***A:*** *Effect of time and training group on emotion regulation* | | | | |
| --- | --- | --- | --- | --- |
|  | **Emotion Regulation** | | | |
| *Predictors* | *df* | *F* | *p* | *R^2^m/R^2^c* |
|  |  |  |  | 0.00/0.65 |
| Time | 211.26 | 3.85 | .023 |  |
| Training group | 283.02 | 0.47 | .495 |  |
| Time x Training group | 209.35 | 3.49 | .032 |  |
| ***B:*** *Effect of time and training group on rumination* | | | | |
|  | **Rumination** | | | |
| *Predictors* | *df* | *F* | *p* | *R^2^m/R^2^c* |
|  |  |  |  | 0.04/0.61 |
| Time | **213.93** | **9.79** | **<.001** |  |
| Training group | 288.48 | 3.22 | .074 |  |
| Time x Training group | 211.50 | 1.14 | .322 |  |
| ***C:*** *Effect of time and training group on depression* | | | | |
|  | **Depression** | | | |
| *Predictors* | *df* | *F* | *p* | *R^2^m/R^2^c* |
|  |  |  |  | 0.02/0.86 |
| Time | 202.71 | 1.82 | .164 |  |
| Training group | 199.86 | 4.43 | .037 |  |
| Time x Training group | 202.11 | 2.58 | .078 |  |
| ***D:*** *Effect of time and training group on anxiety* | | | | |
|  | **Anxiety** | | | |
| *Predictors* | *df* | *F* | *p* | *R^2^m/R^2^c* |
|  |  |  |  | 0.02/0.81 |
| Time | 204.00 | 0.16 | .853 |  |
| Training group | 218.78 | 2.88 | .091 |  |
| Time x Training group | 203.16 | 0.62 | .537 |  |

*Note*. Bolded text indicates statistically significant effects (*p* < .003). **Time** indicates time point of measure completion (baseline vs post-training vs follow-up). **Emotion Regulation** was operationalised as total score on ERQ reappraisal subscale. **Rumination** was operationalised as total score on the RTQ. **Depression** was operationalised as total score on PHQ. **Anxiety** was operationalised as total score on GAD.

**Table S9**

*Summary of H3a Analyses*

| ***A:*** *H3a Analyses: Emotion Regulation* | | | | | | | | |
| --- | --- | --- | --- | --- | --- | --- | --- | --- |
|  | **Emotion Regulation** | | | | | | | |
| *Predictors* | *df* | | *F* | | *p* | | *R^2^m/R^2^c* | |
|  |  | |  | |  | | 0.04/0.66 | |
| Time | 100.29 | | 2.29 | | .133 | |  | |
| Training group | 145.31 | | 3.10 | | .080 | |  | |
| Affective control change | 143.62 | | 0.47 | | .494 | |  | |
| Days since training | 121.50 | | 2.60 | | .109 | |  | |
| Time x Training group | 100.05 | | 3.37 | | .069 | |  | |
| Time x Affective control change | 98.27 | | 0.60 | | .442 | |  | |
| Training group x Affective control change | 144.08 | | 0.27 | | .602 | |  | |
| Time x Training group x Affective control change | 98.31 | | 0.00 | | .972 | |  | |
| ***B:*** *H3a Analyses: Rumination* | | | | | | | | |
|  | | **Rumination** | | | | | | |
| *Predictors* | | *df* | | *F* | | *p* | | *R^2^m/R^2^c* |
|  | |  | |  | |  | | 0.06/0.61 |
| Time | | 101.10 | | 5.04 | | .027 | |  |
| Training group | | 154.63 | | 0.04 | | .848 | |  |
| Affective control change | | 155.34 | | 2.03 | | .156 | |  |
| Days since training | | 125.81 | | 0.54 | | .464 | |  |
| Time x Training group | | 100.59 | | 0.02 | | .898 | |  |
| Time x Affective control change | | 99.58 | | 0.22 | | .640 | |  |
| Training group x Affective control change | | 154.51 | | 2.78 | | .098 | |  |
| Time x Training group x Affective control change | | 99.88 | | 0.97 | | .326 | |  |

*Note*. Bolded text indicates statistically significant effects. **Time** indicates time point of measure completion (baseline vs post-training). **Training group** indicates app training group (SBT vs AffeCT). **Affective control change** was operationalised as change in average RT on correct trials of the emotional minus neutral 2-back task (Gorilla) from baseline to post-training. **Days since training** indicates time between training completion and post task measure completion. **Emotion Regulation** was operationalised as total score on ERQ reappraisal subscale. **Rumination** was operationalised as total score on the RTQ.

**Table S10**

*Summary of H3b Analyses*

| ***A:*** *H3b Analyses:* *Affective Control and Depressive Symptoms* | | | | |
| --- | --- | --- | --- | --- |
|  | **Depression** | | | |
| *Predictors* | *df* | *F* | *p* | *R^2^m/R^2^c* |
|  |  |  |  | 0.04/0.89 |
| Time | 99.30 | 1.01 | .318 |  |
| Training group | 116.75 | 0.80 | .372 |  |
| Affective control change | 116.09 | 0.19 | .666 |  |
| Days since training | 110.18 | 2.51 | .116 |  |
| Time x Training group | 99.66 | 1.31 | .256 |  |
| Time x Affective control change | 99.03 | 0.40 | .527 |  |
| Training group x Affective control change | 116.89 | 0.06 | .810 |  |
| Time x Training group x Affective control change | 99.27 | 0.82 | .368 |  |
| ***B:*** *H3b Analyses:* *Interpretation Bias and Depressive Symptoms* | | | | |
|  | **Depression** | | | |
| *Predictors* | *df* | *F* | *p* | *R^2^m/R^2^c* |
|  |  |  |  | 0.06/0.89 |
| Time | 97.31 | 0.86 | .355 |  |
| Training group | 114.52 | 0.02 | .875 |  |
| Interpretation bias change | 114.37 | 0.13 | .716 |  |
| Days since training | 108.25 | 3.76 | .055 |  |
| Time x Training group | 97.28 | 2.04 | .157 |  |
| Time x Interpretation bias change | 97.26 | 0.44 | .507 |  |
| Training group x Interpretation bias change | 114.43 | 0.82 | .367 |  |
| Time x Training group x Interpretation bias change | 97.65 | 0.05 | .821 |  |
| ***C:*** *H3b Analyses:* *Affective Control and Anxiety Symptoms* | | | | |
|  | **Anxiety** | | | |
| *Predictors* | *df* | *F* | *p* | *R^2^m/R^2^c* |
|  |  |  |  | 0.05/0.81 |
| Time | 99.26 | 0.02 | .880 |  |
| Training group | 126.47 | 1.91 | .170 |  |
| Affective control change | 125.07 | 0.01 | .904 |  |
| Days since training | 114.02 | 3.40 | .068 |  |
| Time x Training group | 99.23 | 0.53 | .468 |  |
| Time x Affective control change | 98.27 | 0.02 | .887 |  |
| Training group x Affective control change | 125.69 | 0.02 | .890 |  |
| Time x Training group x Affective control change | 98.30 | 0.23 | .631 |  |
| ***D:*** *H3b Analyses:* *Interpretation Bias and Anxiety Symptoms* | | | | |
|  | **Anxiety** | | | |
|  |  |  |  | 0.06/0.82 |
| Time | 97.33 | 0.03 | .853 |  |
| Training group | 122.89 | 0.52 | .470 |  |
| Interpretation bias change | 122.47 | 0.34 | .563 |  |
| Days since training | **111.69** | **4.94** | **.028** |  |
| Time x Training group | 96.83 | 0.00 | .992 |  |
| Time x Interpretation bias change | 96.79 | 2.25 | .137 |  |
| Training group x Interpretation bias change | 124.18 | 0.37 | .545 |  |
| Time x Training group x Interpretation bias change | 97.23 | 0.01 | .936 |  |

*Note*. Bolded text indicates statistically significant effects. **Time** indicates time point of measure completion (baseline vs post-training). **Training group** indicates app training group (SBT vs AffeCT). **Affective control change** was operationalised as change in average RT on correct trials of the emotional minus neutral 2-back task (Gorilla) from baseline to post-training. **Interpretation Bias Change** was operationalised as change in proportion of negative grammatically correct sentences in the SST from baseline to post-training. **Days since training** indicates time between training completion and post task measure completion. **Depression** was operationalised as total score on PHQ. **Anxiety** was operationalised as total score on GAD.

**Table S11**

*Means and standard deviations of outcome variables of interest across time points by training group*

| **Variable** | **Baseline**  ***mean (SD)*** | **Post-training**  ***mean (SD)*** | **Follow-up**  ***mean (SD)*** |
| --- | --- | --- | --- |
| **SBT training group** | | | |
| Affective Control | -21.67 (213.42) | 22.06 (215.38) | N/A |
| Interpretation Bias | 0.35 (0.25) | 0.24 (0.22) | N/A |
| Emotion Regulation | 26.09 (6.83) | 27.95 (8.15) | 28.02 (7.35) |
| Rumination | 30.41 (8.86) | 28.15 (9.25) | 27.33 (9.52) |
| Depression | 7.01 (6.46) | 5.95 (6.04) | 6.71 (6.90) |
| Anxiety | 7.24 (6.04) | 5.31 (5.73) | 5.98 (6.30) |
| Social Sensitivity | 26.78 (10.01) | 26.00 (11.29) | 24.18 (9.57) |
| Social Risk Concern | 42.32 (18.51) | 40.46 (17.48) | 39.36 (18.15) |
| Affective Control Change (baseline to post-training) | N/A | -52.11 (248.12) | N/A |
| Interpretation Bias Change (baseline to post-training) | N/A | 0.11 (0.18) | N/A |
| **AffeCT training group** | | | |
| Affective Control | 15.55 (259.66) | -0.27 (176.87) | N/A |
| Interpretation Bias | 0.24 (0.22) | 0.18 (0.20) | N/A |
| Emotion Regulation | 28.21 (5.02) | 28.07 (4.89) | 26.54 (6.41) |
| Rumination | 29.38 (8.76) | 26.83 (9.22) | 25.10 (8.06) |
| Depression | 6.24 (6.81) | 4.11 (5.70) | 4.38 (6.18) |
| Anxiety | 5.54 (6.01) | 4.00 (4.53) | 4.39 (5.40) |
| Social Sensitivity | 23.29 (9.61) | 22.17 (10.70) | 22.28 (8.74) |
| Social Risk Concern | 39.88 (17.57) | 37.72 (14.62) | 39.61 (12.84) |
| Affective Control Change (baseline to post-training) | N/A | 21.83 (220.92) | N/A |
| Interpretation Bias Change (baseline to post-training) | N/A | -0.00 (0.16) | N/A |

*Note*. **Affective Control** = average RT on correct trials of the emotional 2-back task minus average RT on correct trials of the neutral 2-back task. **Interpretation Bias** = proportion of negative grammatically correct sentences in the Scrambled Sentences Task. **Emotion Regulation** = total score on ERQ reappraisal subscale. **Rumination** = total score on RTQ. **Depression** = total score on PHQ. **Anxiety** = total score on GAD. **Social Sensitivity** = total score on O^2^S^3^. **Social Risk Concern** = Average score of HSRQ rating scales. **Affective Control** **Change** = change in average RT on correct trials of the emotional minus neutral 2-back task from baseline to post-training. **Interpretation Bias Change** = Change in proportion of negative grammatically correct sentences in the SST from baseline to post-training.

**Table S12**

*Full sample characteristics (N = 240), means and standard deviations of variables*

|  | ***Mean (SD)/N (%)*** |
| --- | --- |
| **Age (years)** | 14.70 (1.18) |
| **SES** | 2.77 (0.33) |
| **Gender** |  |
| Female | 102 (42.50%) |
| Male | 136 (56.67%) |
| Non-binary | 2 (0.83%) |
| **Country** |  |
| Australia | 114 (47.50%) |
| United Kingdom | 79 (32.92%) |
| United States of America | 45 (18.75%) |
| India | 2 (0.83%) |
| **Ethnicity** |  |
| Aboriginal or Torres Strait Islander | 2 (0.83%) |
| Asian | 27 (11.25%) |
| Black | 29 (12.08%) |
| Hispanic | 1 (0.42%) |
| White | 166 (69.17%) |
| Mixed | 10 (4.17%) |
| Other | 4 (1.67%) |
| Prefer not to say | 1 (0.42%) |
| **Education** |  |
| Current student | 238 (99.17%) |
| Prefer not to say | 2 (0.83%) |
| **Baseline Measures** |  |
| **Affective Control** | -0.86 (203.56) |
| **Interpretation Bias** | 0.31 (0.21) |
| **Emotion Regulation** | 29.64 (6.33) |
| **Rumination** | 32.71 (8.52) |
| **Depression** | 6.40 (6.06) |
| **Anxiety** | 6.75 (5.73) |
| **Social Sensitivity** | 27.77 (9.54) |
| **Social Risk Concern** | 44.96 (17.31) |
| **Post Training Measures** |  |
| **Affective Control** | 11.29 (162.23) |
| **Interpretation Bias** | 0.23 (0.20) |
| **Emotion Regulation** | 30.19 (6.25) |
| **Rumination** | 31.33 (9.13) |
| **Depression** | 5.30 (5.38) |
| **Anxiety** | 5.43 (5.33) |
| **Social Sensitivity** | 27.47 (10.04) |
| **Social Risk Concern** | 43.97 (15.34) |
| **Affective Control Change** | -14.62 (211.85) |
| **Interpretation Bias Change** | 0.06 (0.19) |
| **1-month Follow-up Measures** |  |
| **Emotion Regulation** | 29.45 (6.69) |
| **Rumination** | 31.61 (10.05) |
| **Depression** | 6.84 (6.80) |
| **Anxiety** | 6.87 (6.21) |
| **Social Sensitivity** | 28.15 (9.18) |
| **Social Risk Concern** | 44.20 (14.30) |
| **SBT Training (*n = 124*)** |  |
| **SBT app usage** | 59.73 (67.24) |
| **SBT app sessions** | 6.61 (7.27) |
| **SBT n-back usage** | 80.16 (56.61) |
| **SBT max N** | 3.29 (1.92) |
| **SBT mean N** | 2.01 (1.07) |
| **SBT CBMI usage** | 45.15 (53.48) |
| **SBT CBMI RT** | 3800.96 (599.38) |
| **SBT CBMI accuracy** | 44.63 (34.13) |
| **SBT psychoed usage** | 20.77 (25.31) |
| **AffeCT Training (*n = 116*)** |  |
| **AffeCT app usage** | 40.58 (81.82) |
| **AffeCT app sessions** | 3.62 (7.08) |
| **AffeCT n-back usage** | 102.28 (89.72) |
| **AffeCT max N** | 2.94 (2.49) |
| **AffeCT mean N** | 1.68 (1.13) |

*Note*. **SES** = socio-economic status, derived from the average level of parental education for up to 2 parents (1 = primary school; 2 = high school, professional/vocational training; 3 = university). **Affective Control** = average RT on correct trials of the emotional 2-back task minus average RT on correct trials of the neutral 2-back task. **Interpretation Bias** = proportion of negative grammatically correct sentences in the Scrambled Sentences Task. **Emotion Regulation** = total score on ERQ reappraisal subscale. **Rumination** = total score on RTQ. **Depression** = total score on PHQ. **Anxiety** = total score on GAD. **Social Sensitivity** = total score on O^2^S^3^. **Social Risk Concern** = Average score of HSRQ rating scales. **Affective Control** **Change** = change in average RT on correct trials of the emotional minus neutral 2-back task (Gorilla) from baseline to post-training. **Interpretation Bias Change** = Change in proportion of negative grammatically correct sentences in the SST from baseline to post-training. **App usage** = average time (mins) spent in the assigned training app in total. **App sessions** = average number of training sessions completed in the assigned training app in total. **N-back usage** = amount of time (mins) spent training on the n-back task of the assigned training app. **Max N** = maximum N reached across all trials of the assigned training app. **Mean N** = average N reached across all trials of the assigned training app. **CBMI usage** = total time (mins) spent on CBMI tasks in the SBT app across all SBT app sessions. **CBMI RT** = average RT (ms) on correct CBMI trials across all SBT app sessions. **CBMI accuracy** = total correct CBMI trials across all SBT app sessions. **Psychoed usage** = total time (mins) spent on psychoeducation components in the SBT app across all SBT app sessions.

**Table S13**

*Full Sample Summary of H1 Analyses*

| ***A:*** *H1 Analyses: App Usage* | | | | | |
| --- | --- | --- | --- | --- | --- |
|  | **App Usage** | | | | |
| *Predictors* | *df_regression_* | *df_residual_* | *F* | *p* | *R^2^* |
| Training group | 1 | 238.00 | 3.11 | .079 | 0.01 |
| ***B:*** *H1 Analyses: N-back Usage (exploratory)* | | | | | |
|  | **N-back Usage** | | | | |
| *Predictors* |  | *df* | *F* | *p* | *R^2^* |
| Training group | 1 | 238.00 | 0.66 | .417 | -0.00 |
| ***B:*** *H1 Analyses: Session Number (exploratory)* | | | | | |
|  | **Session Number** | | | | |
| *Predictors* |  | *df* | *F* | *p* | *R^2^* |
| Training group | **1** | **238.00** | **10.17** | **.002** | **0.04** |

*Note*. Bolded text indicates statistically significant effects. **Training group** indicates app training group (SBT vs AffeCT). **App usage** indicates average time (mins) spent in the assigned training app in total. **N-back usage** indicates amount of time (mins) spent training on the n-back task of the assigned training app. **App sessions** indicates average number of training sessions completed in the assigned training app in total.

**Table S14**

*Full Sample Summary of H2 Analyses*

| ***A:*** *H2a Analyses* | | | | |
| --- | --- | --- | --- | --- |
|  | **Affective Control** | | | |
| *Predictors* | *df* | *F* | *p* | *R^2^m/R^2^c* |
|  |  |  |  | 0.01/0.24 |
| Time | 201.00 | 0.37 | .542 |  |
| App usage | 379.36 | 0.27 | .602 |  |
| Days since training | 200.00 | 0.29 | .589 |  |
| Time x App usage | 201.00 | 0.12 | .725 |  |
| ***B:*** *H2b Analyses* | | | | |
|  | **Interpretation Bias** | | | |
| *Predictors* | *df* | *F* | *p* | *R^2^m/R^2^c* |
|  |  |  |  | 0.06/0.57 |
| Time | **199.32** | **8.01** | **.005** |  |
| App usage | **306.28** | **10.94** | **.001** |  |
| Days since training | 199.74 | 1.47 | .227 |  |
| Time x App usage | 197.93 | 2.23 | .137 |  |
| ***C:*** *H2c Analyses* | | | | |
|  | **Interpretation Bias** | | | |
| *Predictors* | *df* | *F* | *p* | *R^2^m/R^2^c* |
|  |  |  |  | 0.08/0.58 |
| Time | 197.21 | 3.26 | .073 |  |
| App usage | 303.62 | 1.86 | .173 |  |
| Training group | 307.69 | 1.49 | .223 |  |
| Days since training | 197.74 | 1.43 | .233 |  |
| Time x App usage | 195.73 | 0.05 | .825 |  |
| Time x Training group | 197.31 | 0.08 | .778 |  |
| App usage x Training group | 305.63 | 1.27 | .262 |  |
| Time x App usage x Training group | 195.93 | 1.10 | .295 |  |

*Note*. Bolded text indicates statistically significant effects. **Time** indicates time point of measure completion (baseline vs post-training). **App usage** indicates average time (mins) spent in the assigned training app in total. **Training group** indicates app training group (SBT vs AffeCT). **Days since training** indicates time between training completion and post task measure completion. **Affective control** was operationalised as average RT on correct trials of the emotional 2-back task minus average RT on correct trials of the neutral 2-back task (measured at baseline and post-training). **Interpretation bias** was operationalised as the proportion of negative grammatically correct sentences in the Scrambled Sentences Task (measured at baseline and post-training).

**Table S15**

*Full Sample Summary of H3 Analyses*

| ***A:*** *H3a Analyses: Emotion Regulation* | | | | |
| --- | --- | --- | --- | --- |
|  | **Emotion Regulation** | | | |
| *Predictors* | *df* | *F* | *p* | *R^2^m/R^2^c* |
|  |  |  |  | 0.06/0.69 |
| Time | **192.75** | **8.82** | **.003** |  |
| Training group | 271.74 | 3.41 | .066 |  |
| Affective control change | 269.64 | 1.15 | .284 |  |
| Days since training | **227.84** | **17.05** | **<.001** |  |
| Time x Training group | **192.47** | **7.20** | **.008** |  |
| Time x Affective control change | 190.68 | 0.10 | .749 |  |
| Training group x Affective control change | 270.86 | 0.03 | .857 |  |
| Time x Training group x Affective control change | 190.88 | 0.08 | .781 |  |
| ***B:*** *H3a Analyses: Rumination* | | | | |
|  | **Rumination** | | | |
| *Predictors* | *df* | *F* | *p* | *R^2^m/R^2^c* |
|  |  |  |  | 0.07/0.60 |
| Time | **191.63** | **11.75** | **.001** |  |
| Training group | 298.62 | 0.13 | .718 |  |
| Affective control change | 296.30 | 0.79 | .375 |  |
| Days since training | **237.26** | **13.81** | **<.001** |  |
| Time x Training group | 191.90 | 1.04 | .310 |  |
| Time x Affective control change | 189.92 | 0.14 | .711 |  |
| Training group x Affective control change | 296.38 | 1.41 | .236 |  |
| Time x Training group x Affective control change | 190.89 | 0.43 | .513 |  |
| ***C:*** *H3b Analyses:* *Affective Control and Depressive Symptoms* | | | | |
|  | **Depression** | | | |
| *Predictors* | *df* | *F* | *p* | *R^2^m/R^2^c* |
|  |  |  |  | 0.02/0.76 |
| Time | 193.10 | 0.38 | .537 |  |
| Training group | 251.95 | 1.61 | .206 |  |
| Affective control change | 250.20 | 0.08 | .778 |  |
| Days since training | 221.49 | 1.02 | .314 |  |
| Time x Training group | 193.59 | 0.06 | .803 |  |
| Time x Affective control change | 192.22 | 0.76 | .384 |  |
| Training group x Affective control change | 251.65 | 0.25 | .616 |  |
| Time x Training group x Affective control change | 192.69 | 1.13 | .288 |  |
| ***D:*** *H3b Analyses:* *Interpretation Bias and Depressive Symptoms* | | | | |
|  | **Depression** | | | |
| *Predictors* | *df* | *F* | *p* | *R^2^m/R^2^c* |
|  |  |  |  | 0.02/0.76 |
| Time | 188.99 | 0.17 | .680 |  |
| Training group | 246.53 | 0.46 | .500 |  |
| Interpretation bias change | 246.96 | 0.00 | .946 |  |
| Days since training | 217.27 | 1.39 | .239 |  |
| Time x Training group | 188.80 | 0.05 | .816 |  |
| Time x Interpretation bias change | 188.74 | 0.06 | .800 |  |
| Training group x Interpretation bias change | 246.53 | 1.58 | .210 |  |
| Time x Training group x Interpretation bias change | 189.90 | 0.31 | .576 |  |
| ***E:*** *H3b Analyses:* *Affective Control and Anxiety Symptoms* | | | | |
|  | **Anxiety** | | | |
| *Predictors* | *df* | *F* | *p* | *R^2^m/R^2^c* |
|  |  |  |  | 0.01/0.75 |
| Time | 192.59 | 1.68 | .196 |  |
| Training group | 253.88 | 0.08 | .779 |  |
| Affective control change | 251.04 | 0.16 | .686 |  |
| Days since training | 220.99 | 0.16 | .686 |  |
| Time x Training group | 192.80 | 0.09 | .765 |  |
| Time x Affective control change | 191.53 | 0.09 | .759 |  |
| Training group x Affective control change | 252.40 | 0.37 | .546 |  |
| Time x Training group x Affective control change | 191.71 | 0.16 | .691 |  |
| ***F:*** *H3b Analyses:* *Interpretation Bias and Anxiety Symptoms* | | | | |
|  | **Anxiety** | | | |
|  |  |  |  | 0.02/0.75 |
| Time | 188.92 | 0.67 | .413 |  |
| Training group | 248.31 | 0.00 | .957 |  |
| Interpretation bias change | 246.69 | 1.78 | .184 |  |
| Days since training | 216.78 | 0.48 | .488 |  |
| Time x Training group | 188.48 | 0.18 | .669 |  |
| Time x Interpretation bias change | 187.88 | 1.73 | .190 |  |
| Training group x Interpretation bias change | 250.56 | 0.20 | .658 |  |
| Time x Training group x Interpretation bias change | 188.95 | 0.31 | .576 |  |

*Note*. Bolded text indicates statistically significant effects. **Time** indicates time point of measure completion (baseline vs post-training). **Training group** indicates app training group (SBT vs AffeCT). **Affective control change** was operationalised as change in average RT on correct trials of the emotional minus neutral 2-back task (Gorilla) from baseline to post-training. **Interpretation Bias Change** was operationalised as change in proportion of negative grammatically correct sentences in the SST from baseline to post-training. **Days since training** indicates time between training completion and post task measure completion. **Emotion Regulation** was operationalised as total score on ERQ reappraisal subscale. **Rumination** was operationalised as total score on the RTQ. **Depression** was operationalised as total score on PHQ. **Anxiety** was operationalised as total score on GAD.
